# Comparative efficacy and safety of repositioning maneuvers for posterior canal benign paroxysmal positional vertigo: a network meta-analysis

**DOI:** 10.3389/fneur.2026.1762375

**Published:** 2026-02-02

**Authors:** Hong Xin, Ningning Fang, Mengmeng Wu

**Affiliations:** 1 Department of Neurology (Ward 4), Binzhou People's Hospital, Binzhou, Shandong, China; 2 Day Ward, Binzhou People's Hospital, Binzhou, Shandong, China; 3 The Second Ward of Pulmonary and Critical Care Medicine Department, Binzhou People's Hospital, Binzhou, Shandong, China

**Keywords:** benign paroxysmal positional vertigo, canalith-repositioning maneuver, Epley maneuver, network meta-analysis, posterior canal, Semont maneuver

## Abstract

**Objective:**

This study aimed to systematically evaluate and compare the efficacy and safety of different repositioning maneuvers for posterior canal benign paroxysmal positional vertigo (BPPV).

**Methods:**

PubMed, Embase, Web of Science and the Cochrane Library were searched from inception to August 2025. Randomized controlled trials (RCTs) comparing the Epley, Semont, Brandt–Daroff, and other repositioning maneuvers for posterior canal BPPV were included. Two reviewers independently performed literature screening, data extraction and risk-of-bias assessment. Network meta-analysis and league tables were generated using StataSE 15 and R 4.4.3, respectively.

**Results:**

Twenty RCTs involving 2,089 patients were included. The Epley maneuver ranked highest in overall effectiveness, with a surface under the cumulative ranking curve (SUCRA) of 97.84%, and was significantly superior to the Semont maneuver (RR = 1.04), the Brandt–Daroff maneuver (RR = 1.35) and control (RR = 1.30). For cure rate, the Epley and Semont maneuvers performed best and were significantly more effective than other interventions. For recurrence rate, the quality of evidence was generally low, and no optimal strategy could be identified. For safety, the incidence of nausea, vomiting and dizziness showed no statistically significant differences among the Epley, Semont and Brandt–Daroff maneuvers, although SUCRA rankings indicated a more favorable safety profile for the Epley maneuver.

**Conclusion:**

The Epley and Semont maneuvers demonstrated optimal short-term efficacy (effectiveness and cure rate) with favorable safety profiles for posterior canal BPPV and should be recommended as first-line repositioning strategies. Future high-quality studies are needed to clarify the long-term effects on recurrence and applicability in specific populations.

**Systematic review registration:**

https://www.crd.york.ac.uk/PROSPERO, identifier CRD420250653366.

## 1 Background

Benign paroxysmal positional vertigo is characterized by transient episodes of vertigo triggered by position-specific head movements and accounts for approximately 60% of peripheral vertigo cases, with an incidence of approximately 64 per 10,000 individuals (1). The incidence peaks between 50 and 70 years of age, with women exhibiting a two-fold higher incidence than men (2). Although classified as a benign disorder, BPPV substantially impairs quality of life, functional status and psychological well-being. Typical vertigo episodes frequently cause balance impairment and increase fall risk, leading to activity restriction and reduced independence. Unpredictable symptoms often induce marked anxiety and fear, directly interfering with daily activities, occupational performance and social participation (3). BPPV therefore is not merely a common sensory disturbance but a significant health concern that compromises physical and psychological health as well as social functioning.

Among the clinical subtypes of BPPV, the most prevalent form results from freely floating otoconial debris within the posterior semicircular canal (canalithiasis), accounting for the vast majority of cases (4). For this subtype, canalith-repositioning maneuvers constitute the cornerstone of treatment. These maneuvers guide displaced otoconia (calcium carbonate crystals) from the affected semicircular canal back into the utricle through a sequence of specific head and body positions that utilize gravitational forces, thereby eliminating symptoms at their source (5). The selection of a specific maneuver depends on the BPPV subtype and the anatomical location of the otoconial debris. Various maneuvers, including the Epley, Semont, Brandt–Daroff, and Gans maneuvers, are widely employed in clinical practice (4), each with distinctive procedural features and theoretical advantages.

Although repositioning maneuvers have proven effective, debates persist regarding the relative efficacy and safety of different techniques. Existing systematic reviews and meta-analyses have predominantly compared the Epley and Semont maneuvers or contrasted single maneuvers with sham or pharmacological interventions (6). Direct comparative evidence for key self-administered maneuvers such as the Epley, Semont and traditional Brandt–Daroff maneuvers remains limited, and comprehensive evidence synthesis is lacking (7). Determining which self-repositioning strategy offers the optimal benefit-risk balance is a critical question in clinical practice.

Given these evidence gaps, this study aimed to systematically evaluate and compare the clinical efficacy and adverse events of the Epley, Semont, Brandt–Daroff and other commonly used self-repositioning maneuvers for BPPV through systematic review and network meta-analysis, with the goal of providing the highest level of evidence-based guidance for clinicians and patients in selecting optimal individualized treatment strategies.

## 2 Methods

This study was conducted following the Preferred Reporting Items for Systematic Reviews and Meta-Analyses extension for Network Meta-Analyses (PRISMA-NMA) guidelines (8). The protocol was registered in PROSPERO (CRD420250653366).

### 2.1 Search strategy

A systematic search combining controlled vocabulary and free-text terms was performed in PubMed, Embase, Web of Science and the Cochrane Library. The search covered records from database inception to August 2025. Search terms included BPPV, repositioning maneuver and posterior canal. Medical Subject Headings (MeSH) and Emtree terms were combined with free-text words using Boolean operators (AND, OR and NOT) to construct the search strategy, which is presented in Supplementary Table S1.

### 2.2 Inclusion criteria

The inclusion criteria were defined according to the PICOS framework. The target population comprised patients diagnosed with BPPV. Interventions included the Epley, Semont, Brandt–Daroff, and Gans maneuvers. Comparators consisted of sham procedures, observation controls or alternative repositioning maneuvers such as the Particle Repositioning Maneuver (PRM). Outcomes included effectiveness, cure rate, recurrence rate and adverse events including nausea, vomiting and dizziness. Effectiveness was defined as clinical improvement without complete symptom resolution after treatment. Cure rate was defined as complete resolution of symptoms and signs following the repositioning maneuver. Recurrence rate was defined as the proportion of patients experiencing reappearance of symptoms and signs during a defined follow-up period after initial cure. Only randomized controlled trials (RCTs) were included.

### 2.3 Exclusion criteria

The exclusion criteria were as follows: animal experiments, *in vitro* studies, computer simulation studies, reviews, systematic reviews, meta-analyses, conference abstracts, expert opinions and other non-clinical research; studies that did not report key outcome indicators (such as symptom relief rate) or for which full texts were inaccessible; trials combining other therapies (such as medication with repositioning maneuvers) without separately analyzing the effects of repositioning maneuvers. When duplicate publications from the same dataset were identified across journals, only the version with the most complete data was included.

### 2.4 Literature screening and data extraction

Two investigators, Hong Xin and Ningning Fang, independently performed literature screening in EndNote according to the predefined eligibility criteria. Titles and abstracts were reviewed, and potentially eligible full texts were retrieved and evaluated. Disagreements were resolved through discussion with a third investigator, Mengmeng Wu. The same two investigators independently extracted data including author name, publication year, region, sample size, intervention, patient age, sex, number of responders, recurrence rate, cure rate and the incidence of adverse events (nausea, vomiting and dizziness). Discrepancies were resolved through consultation with the third investigator.

### 2.5 Quality assessment

Quality assessment was performed by Hong Xin and Ningning Fang using the Risk of Bias 2.0 (RoB 2.0) tool, which comprises five domains evaluating potential sources of bias in RCTs: bias arising from the randomization process, bias due to deviations from intended interventions, bias due to missing outcome data, bias in outcome measurement and bias in selection of reported results (9). Disagreements were resolved through discussion with Mengmeng Wu. The certainty of evidence was evaluated using the GRADE approach, which classifies evidence quality into four levels: high (⊕⊕⊕⊕), moderate (⊕⊕⊕O), low (⊕⊕OO) and very low (⊕OOO). Disagreements in grading were resolved through discussion with the third investigator.

### 2.6 Statistical analysis

Statistical analysis employed an integrated frequentist and Bayesian framework. StataSE 15 was used to generate the network plot depicting relationships among interventions, visually illustrating the structure of available direct comparisons. In the network diagram, nodes represented different interventions, with node size proportional to the sample size for each intervention. Lines between nodes represented direct comparisons, with line thickness proportional to the number of supporting studies. All network meta-analysis models were fitted in R (version 4.4.3) using the gemtc package under a Bayesian framework. To ensure stability and accuracy of parameter estimation, adequate Markov chain Monte Carlo (MCMC) simulation was performed using four independent chains with 20,000 iterations each; the initial 5,000 iterations were discarded as burn-in to ensure convergence. Network-wide heterogeneity was assessed using the generalized *I*^2^ statistic. Meta-regression analyses were conducted to explore potential sources of heterogeneity by evaluating the influence of publication year, mean sample size, mean patient age and proportion of male patients on the primary outcomes (effectiveness, cure rate and recurrence rate). Inconsistency within each closed loop was examined using the node-splitting method, with a Bayesian *P* value < 0.05 indicating significant discrepancy between direct and indirect evidence. Pairwise comparisons among all interventions were presented as risk ratios (RRs) with 95% credible intervals (CrIs) in league tables. Surface under the cumulative ranking curve (SUCRA) values were calculated to rank the relative efficacy of interventions. Funnel plots were constructed to assess potential publication bias. The GRADE framework for network meta-analysis was applied to determine the certainty of evidence for key outcomes.

Surface under the cumulative ranking curve (SUCRA) values were used to rank interventions in this study. Notably, SUCRA values reflect only the relative ranking order of interventions within the existing evidence network; they do not indicate the magnitude of effect sizes or the certainty of evidence. Ranking results should therefore be interpreted in conjunction with pairwise risk ratio (RR) estimates and their credible intervals, as well as GRADE certainty ratings.

## 3 Results

### 3.1 Literature screening

**Figure 1:**
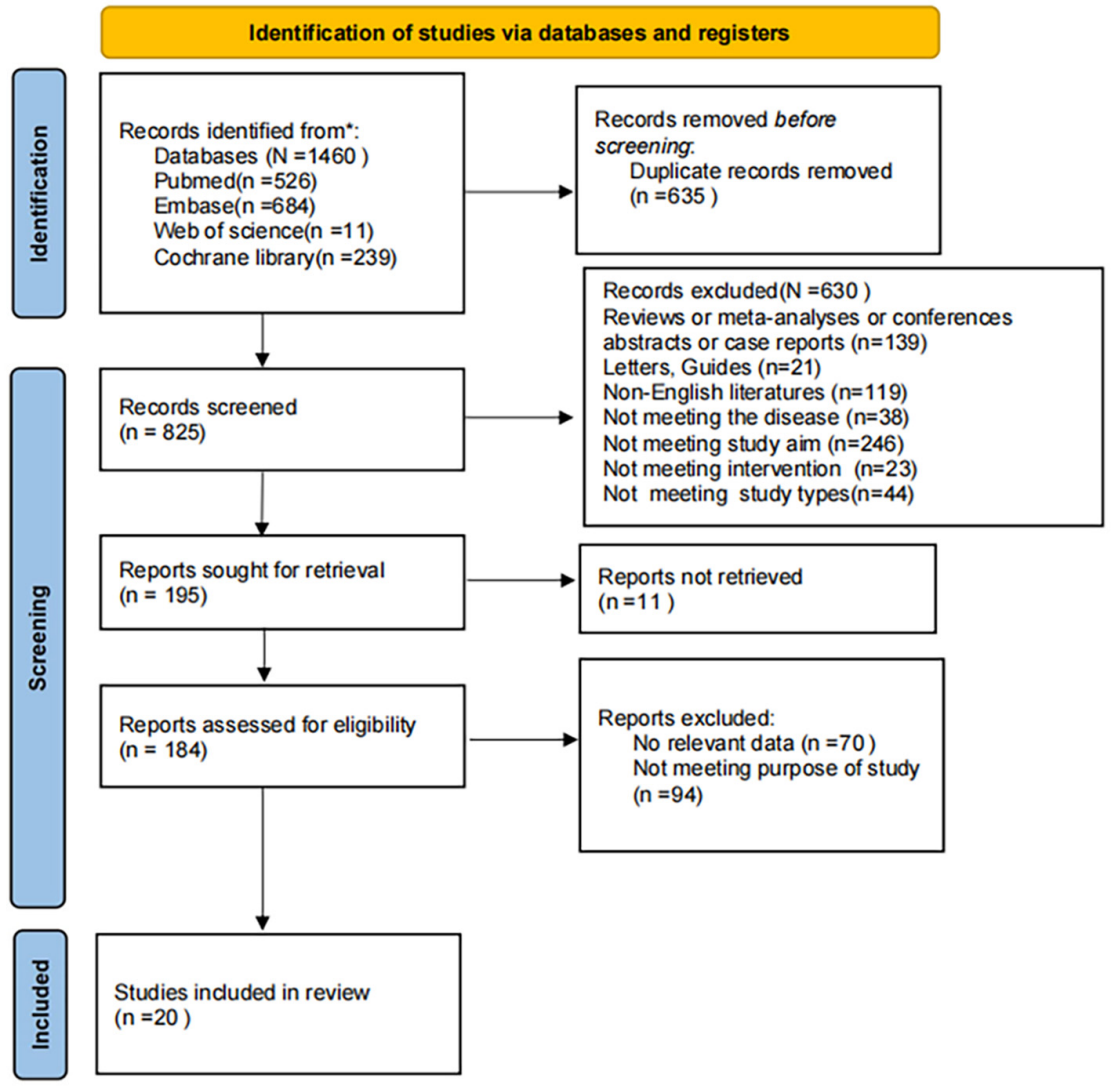
PRISMA flow diagram of literature selection.

The initial search yielded 1,460 records. After removing 635 duplicates, 630 articles were excluded based on title and abstract screening, and 11 were excluded due to unavailable full text. Following full-text review, 164 additional articles were excluded, resulting in 20 studies included in the final analysis. The screening process is shown in Figure 1.

### 3.2 Baseline characteristics

Twenty studies (4, 10–28) involving 2,089 patients were included. The studies were conducted in Germany, South Korea, India, North Korea and the United States. Patient ages ranged from 26 to 65 years, and follow-up duration ranged from 1 week to 1 month. Interventions included the Epley, Semont, Brandt–Daroff and Gans maneuvers. Detailed baseline characteristics are presented in Supplementary Table S2.

### 3.3 Quality assessment

**Figure 2:**
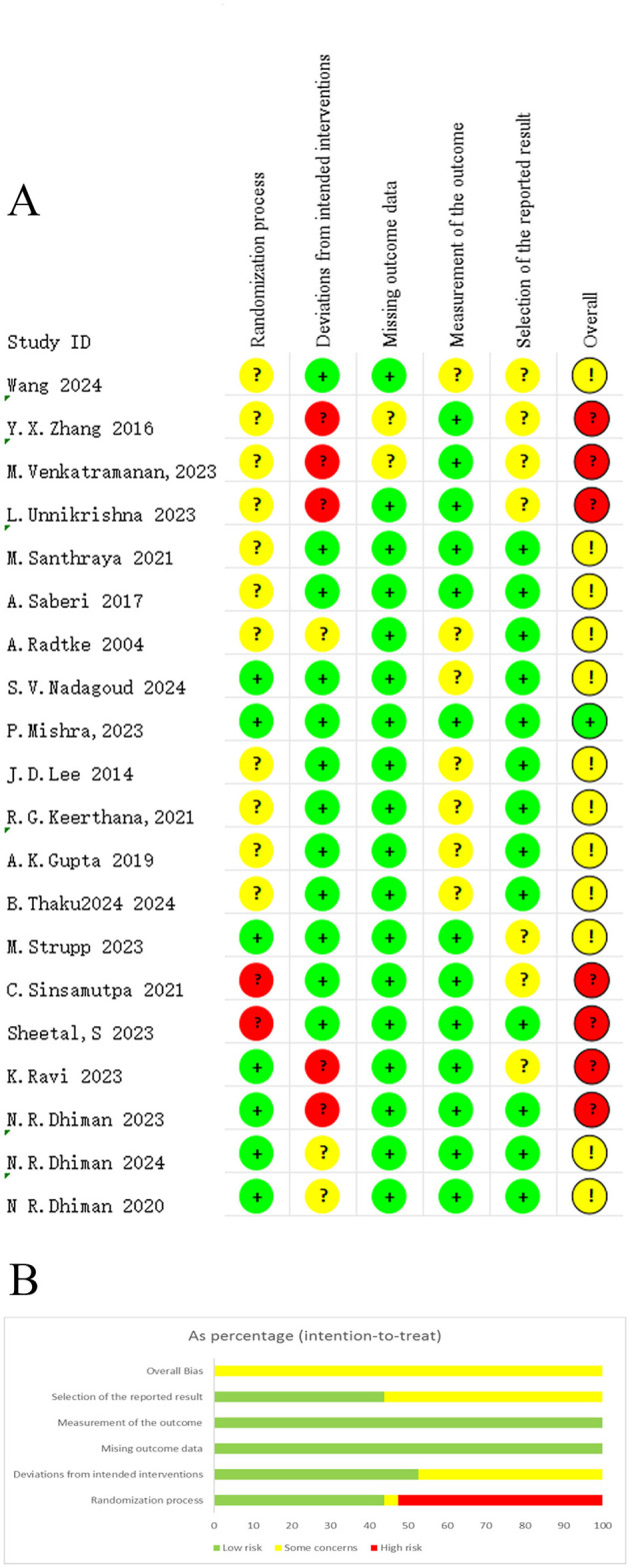
Risk-of-bias summary, **(A)** risk of bias graph; **(B)** summary of risk of bias.

For quality assessment, seven studies were at high risk of bias, one was at low risk and twelve had some concerns. The primary sources of high risk of bias were inadequate randomization procedures and incomplete outcome data. Among studies with some concerns, essential methodological details were insufficiently reported, limiting accurate evaluation of potential bias and compromising reproducibility and certainty of findings. Assessment results are summarized in Figure 2.

### 3.4 Network plots

**Figure 3:**
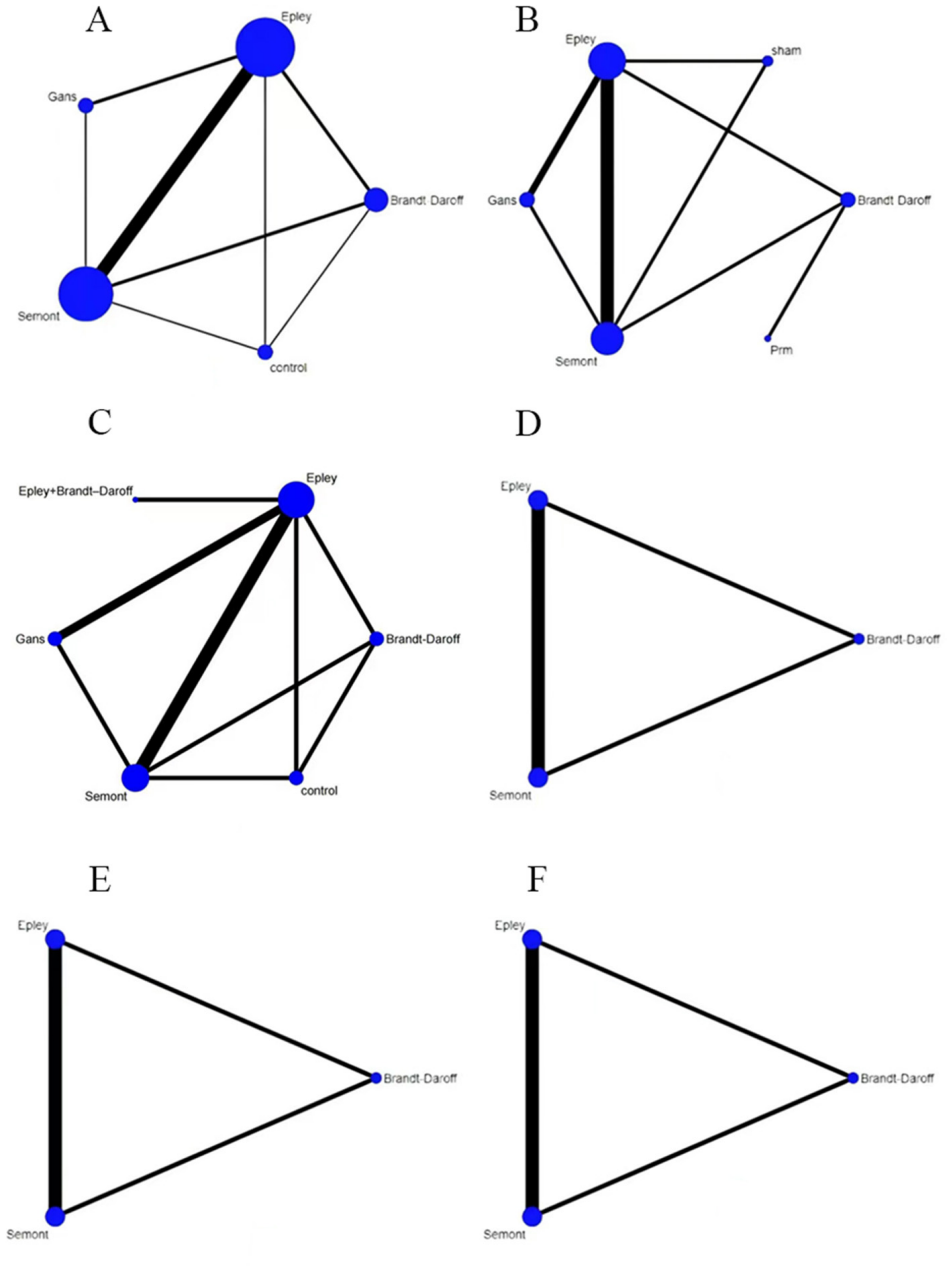
Network plots for outcomes, **(A)** effectiveness; **(B)** cure rate; **(C)** recurrence rate; **(D)** nausea; **(E)** vomiting; **(F)** dizziness.

Network plots were generated using Stata SE 15. Node size represented sample size, and line thickness represented the number of studies supporting each direct comparison. Network structures for each outcome are shown in Figure 3.

### 3.5 Network meta-analysis

#### 3.5.1 Effectiveness

For effectiveness, as shown in Table 1, the Epley maneuver showed significantly higher effectiveness than the Semont maneuver (RR = 1.04, 95% CrI: 1.00–1.11), Brandt–Daroff exercises (RR = 1.35, 95% CrI: 1.17–1.63) and control (RR = 1.30, 95% CrI: 1.12–1.60). The Semont maneuver also demonstrated higher effectiveness than Brandt–Daroff exercises (RR = 1.29, 95% CrI: 1.11–1.56) and control (RR = 1.24, 95% CrI: 1.07–1.54).

**Table 1:** League table for effectiveness.

**Treatment**	**Epley**	**Semont**	**Brandt–Daroff**	**Gans**	**Control**
Epley	NA	0.96 (0.90, 1.00)	0.74 (0.61, 0.85)	0.85 (0.67, 1.04)	0.77 (0.62, 0.89)
		⊕⊕⊕O Moderate	⊕OOO Very Low	⊕⊕⊕O Moderate	⊕⊕⊕O Moderate
Semont	1.04 (1.00, 1.11)	NA	0.77 (0.64, 0.90)	0.89 (0.70, 1.09)	0.81 (0.65, 0.94)
	⊕⊕⊕O Moderate		⊕⊕⊕O Moderate	⊕⊕OO Low	⊕⊕⊕O Moderate
Brandt–Daroff	1.35 (1.17, 1.63)	1.29 (1.11, 1.56)	NA	1.16 (0.88, 1.51)	1.04 (0.81, 1.32)
	⊕OOO Very Low	⊕⊕⊕O Moderate		⊕OOO Very low	⊕⊕OO Low
Gans	1.17 (0.97, 1.49)	1.12 (0.92, 1.42)	0.86 (0.66, 1.14)	NA	0.90 (0.68, 1.19)
	⊕⊕⊕O Moderate	⊕⊕OO Low	⊕OOO Very low		⊕OOO Very low
Control	1.30 (1.12,1.60)	1.24 (1.07, 1.54)	0.96 (0.76, 1.23)	1.11 (0.84, 1.47)	NA
	⊕⊕⊕O Moderate	⊕⊕⊕O Moderate	⊕⊕OO Low	⊕OOO Very low

Evidence quality: high (⊕⊕⊕⊕), moderate (⊕⊕⊕O), low (⊕⊕OO), very low (⊕OOO).

**Figure 4:**
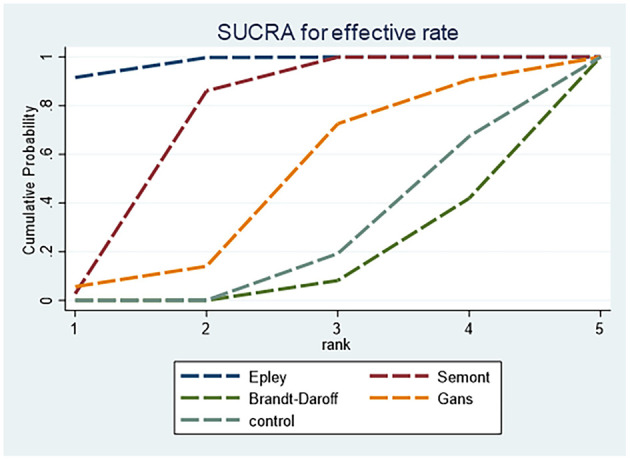
SUCRA ranking curve for effectiveness.

Figure 4 presents the SUCRA rankings for effectiveness: Epley (97.84%) > Semont (72.20%) > Gans (45.73%) > control (21.69%) > Brandt–Daroff (12.53%). The Epley maneuver had the highest SUCRA value, indicating optimal effectiveness.

#### 3.5.2 Cure rate

For cure rate, as shown in Table 2, the Epley maneuver demonstrated significantly higher cure rates than the Semont maneuver (RR = 1.09, 95% CrI: 1.03–1.17), Brandt–Daroff exercises (RR = 1.18, 95% CrI: 1.06–1.41) and control (RR = 1.30, 95% CrI: 1.12–1.60). The Semont maneuver showed higher cure rates than control (RR = 1.19, 95% CrI: 1.02–1.48). Evidence quality for Epley vs. Semont comparisons was predominantly moderate. One study directly compared PRM with Brandt–Daroff exercises and demonstrated superior effectiveness of PRM.

**Table 2:** League table for cure rate.

**Treatment**	**Epley**	**Semont**	**Brandt–Daroff**	**Control**	**Gans**	**Sham**	**PRM**
Epley	NA	0.92 (0.86, 0.97)	0.85 (0.71, 0.95)	0.77 (0.63, 0.89)	0.97 (0.87, 1.09)	0.87 (0.67, 1.07)	2.79 (1.65, 5.43)
		⊕⊕⊕O Moderate	⊕⊕⊕⊕ High	⊕⊕⊕⊕ High	⊕⊕⊕O Moderate	⊕⊕⊕O Moderate	⊕OOO Very low
Semont	1.09 (1.03, 1.17)	NA	0.92 (0.77, 1.05)	0.84 (0.68, 0.98)	1.06 (0.93, 1.21)	0.95 (0.73, 1.17)	3.05 (1.79, 5.94)
	⊕⊕⊕O Moderate		⊕⊕⊕OO Low	⊕⊕⊕⊕ High	⊕⊕OO Low	⊕⊕OO Low	⊕OOO Very low
Brandt–Daroff	1.18 (1.06, 1.41)	1.09 (0.95, 1.3)	NA	0.91 (0.72, 1.13)	1.15 (0.98, 1.42)	1.03 (0.77, 1.35)	3.32 (2.02, 6.37)
	⊕⊕⊕⊕ High	⊕⊕OO Low		⊕⊕OO Low	⊕⊕OO Low	⊕⊕OO Low	⊕⊕⊕O Moderate
Control	1.3 (1.12, 1.6)	1.19 (1.02, 1.48)	1.09 (0.88, 1.38)	NA	1.27 (1.05, 1.6)	1.13 (0.84, 1.52)	3.65 (2.1, 7.28)
	⊕⊕⊕⊕ High	⊕⊕⊕⊕ High	⊕⊕OO Low		⊕⊕OO Low	⊕⊕OO Low	⊕OOO Very low
Gans	1.03 (0.92, 1.15)	0.94 (0.83, 1.07)	0.87 (0.71, 1.02)	0.79 (0.63, 0.95)	NA	0.89 (0.67, 1.13)	2.87 (1.67, 5.63)
	⊕⊕⊕O Moderate	⊕⊕OO Low	⊕⊕OO Low	⊕⊕OO Low		⊕⊕OO Low	⊕OOO Very low
Sham	1.15 (0.93, 1.5)	1.05 (0.86, 1.37)	0.97 (0.74, 1.29)	0.88 (0.66, 1.2)	1.12 (0.88, 1.49)	NA	3.23 (1.81, 6.54)
	⊕⊕⊕O Moderate	⊕⊕OO Low	⊕⊕OO Low	⊕⊕OO Low	⊕⊕OO Low		⊕OOO Very low
PRM	0.36 (0.18, 0.61)	0.33 (0.17, 0.56)	0.3 (0.16, 0.5)	0.27 (0.14, 0.48)	0.35 (0.18, 0.6)	0.31 (0.15, 0.55)	NA
	⊕OOO Very low	⊕OOO Very low	⊕⊕⊕O Moderate	⊕OOO Very low	⊕OOO Very low	⊕OOO Very low	

Evidence quality: high (⊕⊕⊕⊕), moderate (⊕⊕⊕O), low (⊕⊕OO), very low (⊕OOO).

**Figure 5:**
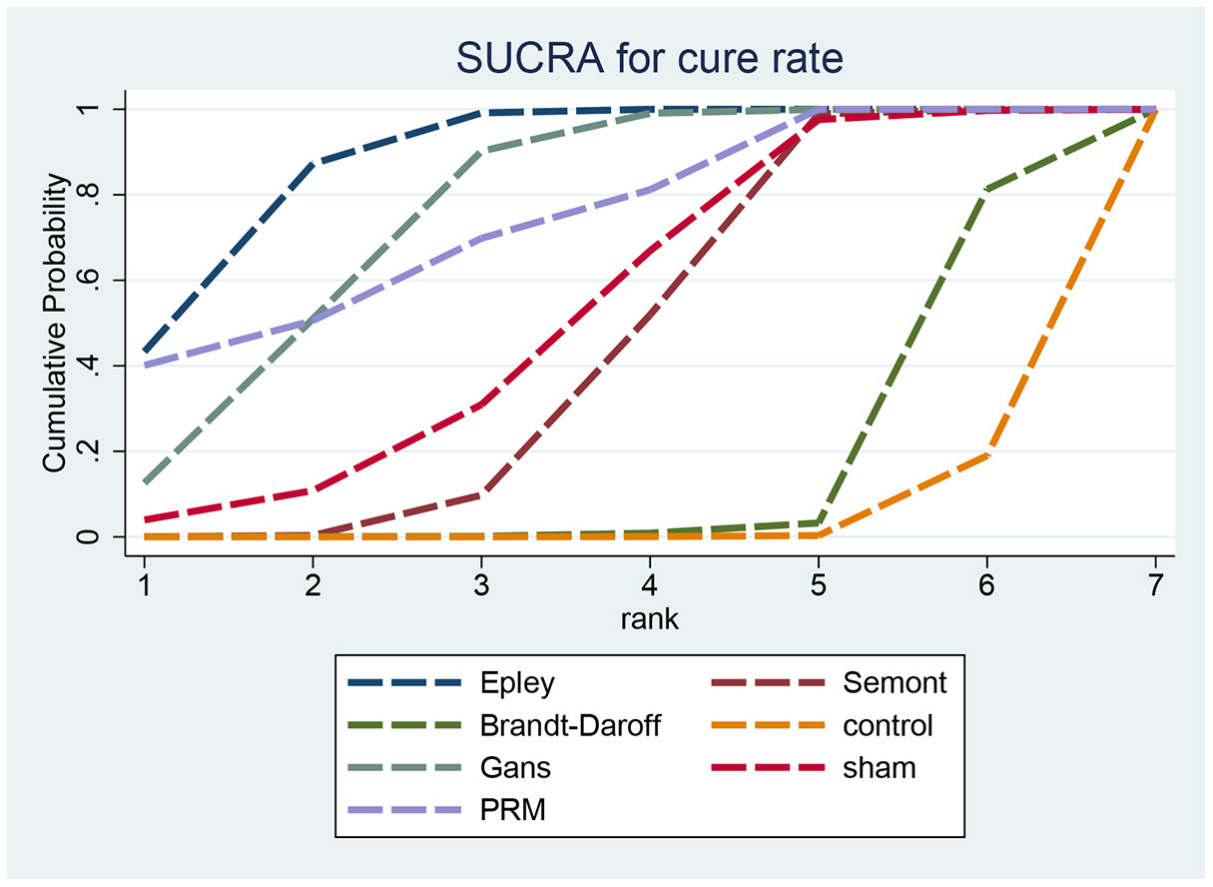
SUCRA ranking curve for cure rate.

Figure 5 presents the SUCRA rankings for cure rate: Epley (76.18%) > Gans (65.01%) > Semont (45.38%) > sham (33.63%) > Brandt–Daroff (22.73%) > control (7.04%). The Epley maneuver showed the highest SUCRA value, indicating optimal efficacy for cure rate.

#### 3.5.3 Recurrence rate

For recurrence rate, as shown in Table 3, the combined Epley plus Brandt–Daroff strategy reduced recurrence compared with Brandt–Daroff alone (RR = 0.21, 95% CrI: 0.05–0.78) and control (RR = 0.23, 95% CrI: 0.06–0.90). Most evidence for recurrence was of low or very low quality, resulting in high uncertainty. Current data do not permit firm conclusions regarding which intervention is superior for reducing recurrence.

**Table 3:** League table for recurrence rate.

**Treatment**	**Epley**	**Semont**	**Brandt–Daroff**	**Control**	**Gans**	**Epley + Brandt–Daroff**
Epley	NA	1.52 (0.67, 3.52)	2.63 (0.96, 7.23)	2.32 (0.84, 6.51)	0.93 (0.31, 2.66)	0.55 (0.22, 1.32)
		⊕⊕OO Low	⊕OOO Very low	⊕OOO Very low	⊕OOO Very low	⊕OOO Very low
Semont	0.66 (0.28, 1.5)	NA	1.73 (0.66, 4.57)	1.53 (0.57, 4.08)	0.61 (0.18, 1.97)	0.36 (0.1, 1.19)
	⊕⊕OO Low		⊕⊕OO Low	⊕⊕OO Low	⊕OOO Very low	⊕OOO Very low
Brandt–Daroff	0.38 (0.14, 1.04)	0.58 (0.22, 1.52)	NA	0.88 (0.33, 2.39)	0.35 (0.09, 1.41)	0.21 (0.05, 0.78)
	⊕OOO Very low	⊕⊕OO Low		⊕⊕OO Low	
Control	0.43 (0.15, 1.2)	0.65 (0.25, 1.75)	1.13 (0.42, 3.03)	NA	0.4 (0.1, 1.6)	0.23 (0.06, 0.9)
	⊕OOO Very low	⊕⊕OO Low	⊕⊕OO Low		⊕OOO Very low	⊕OOO Very low
Gans	1.07 (0.38, 3.27)	1.63 (0.51, 5.59)	2.85 (0.71, 11.6)	2.5 (0.63, 10.43)	NA	0.59 (0.14, 2.42)
	⊕OOO Very low	⊕OOO Very low	⊕OOO Very low	⊕OOO Very low		⊕OOO Very low
Epley + Brandt–Daroff	1.83 (0.76, 4.54)	2.78 (0.84, 9.58)	4.83 (1.28, 18.71)	4.27 (1.11, 16.67)	1.71 (0.41, 6.93)	NA
	⊕OOO Very low	⊕OOO Very low	⊕OOO Very low	⊕OOO Very low	⊕OOO Very low	

Evidence quality: high (⊕⊕⊕⊕), moderate (⊕⊕⊕O), low (⊕⊕OO), very low (⊕OOO).

**Figure 6:**
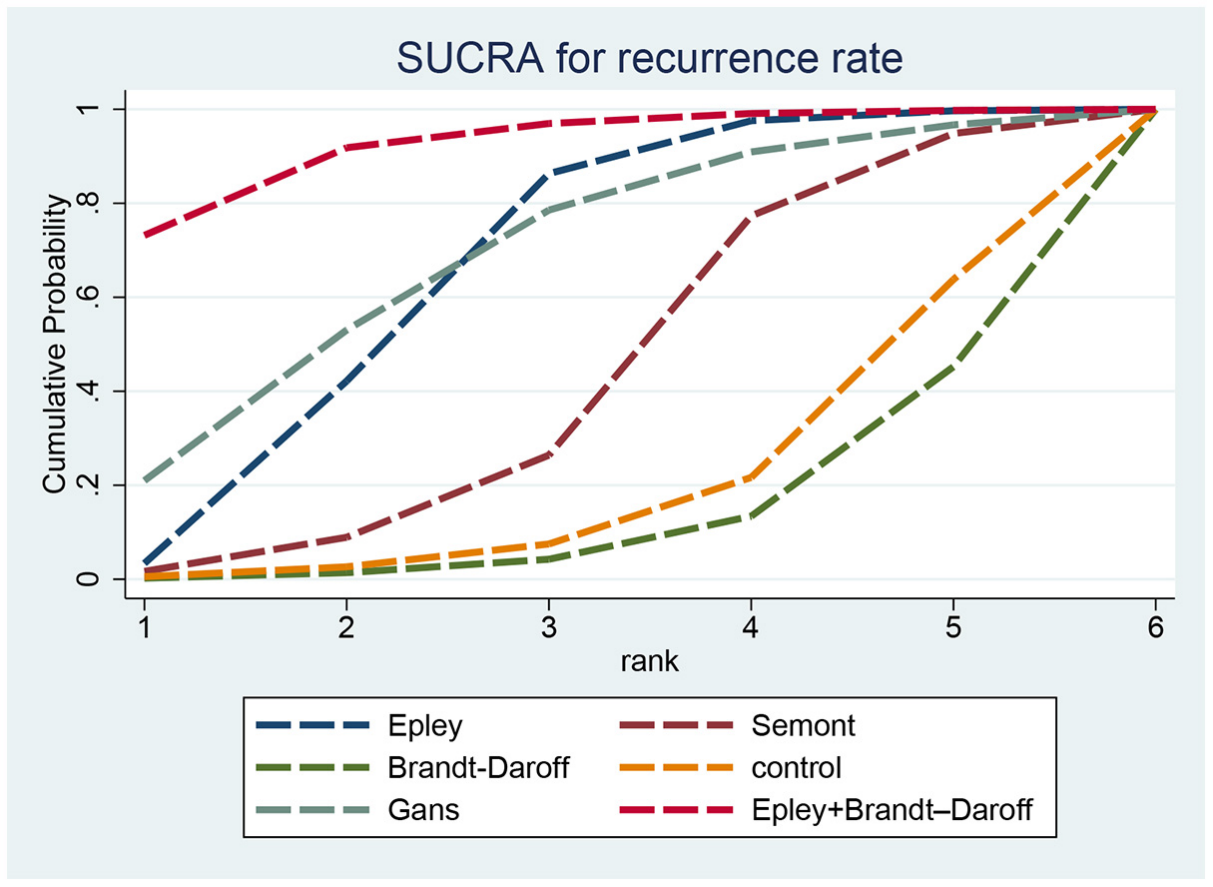
SUCRA ranking curve for recurrence rate.

Figure 6 presents the SUCRA rankings for reducing recurrence: Epley plus Brandt–Daroff (92.17%) > Gans (68.04%) > Epley (65.83%).

#### 3.5.4 Adverse events

**Figure 7:**
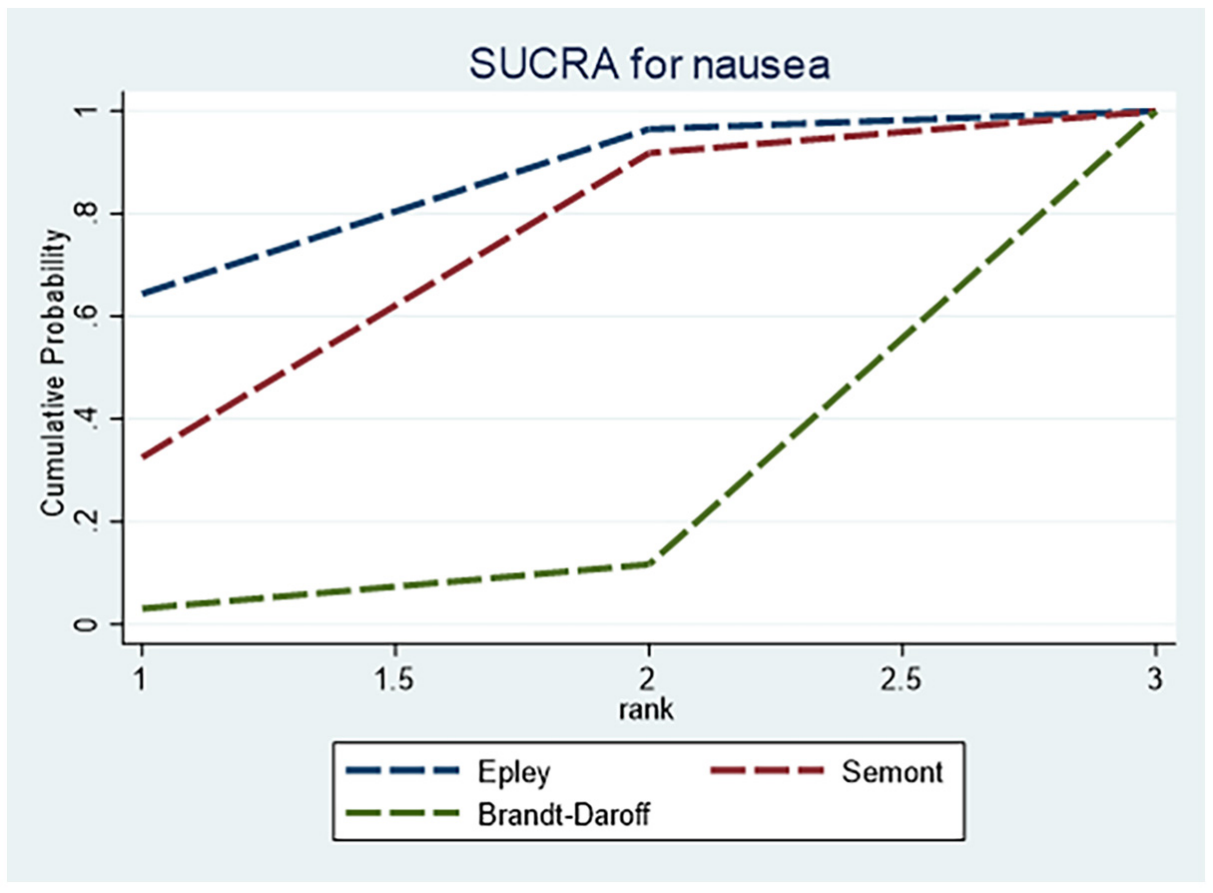
SUCRA ranking curve for nausea.

For adverse events related to nausea, Table 4 shows no statistically significant differences among the Epley, Semont and Brandt–Daroff maneuvers. SUCRA rankings (Figure 7) suggested that the Epley maneuver was least likely to induce nausea, whereas Brandt–Daroff carried a comparatively higher risk.

**Table 4:** League table for nausea.

**Treatment**	**Epley**	**Semont**	**Brandt–Daroff**
Epley	NA	1.08 (0.75, 1.55)	1.44 (0.92, 2.21)
		⊕⊕OO Low	⊕⊕OO Low
Semont	0.93 (0.64, 1.33)	NA	1.33 (0.87, 2.02)
	⊕⊕OO Low		⊕⊕OO Low
Brandt–Daroff	0.70 (0.45, 1.09)	0.75 (0.50, 1.15)	NA
	⊕⊕OO Low	⊕⊕OO Low	

Evidence quality: high (⊕⊕⊕⊕), moderate (⊕⊕⊕O), low (⊕⊕OO), very low (⊕OOO).

**Figure 8:**
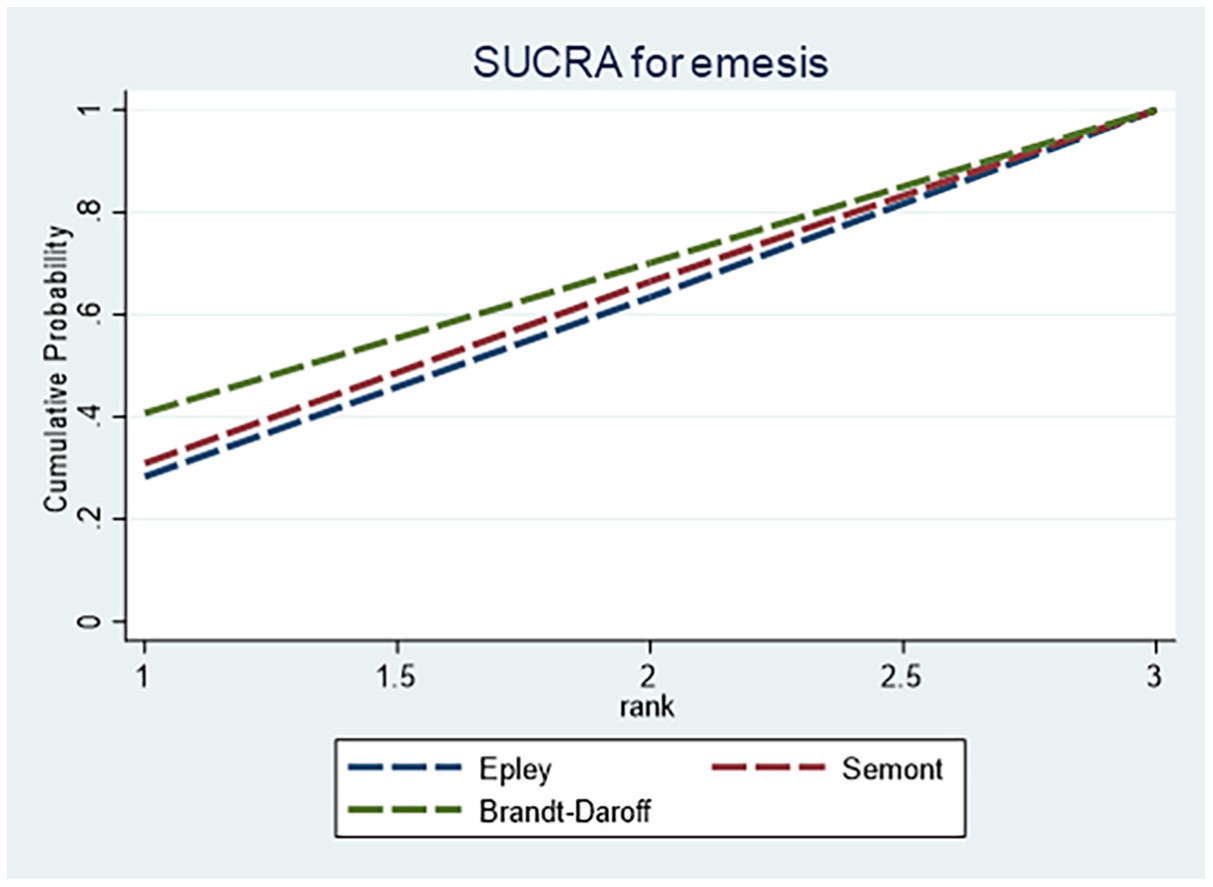
SUCRA ranking curve for vomiting.

For vomiting, Table 5 shows no statistically significant differences among the Epley, Semont and Brandt–Daroff maneuvers. SUCRA rankings (Figure 8) indicated that the Epley maneuver was least likely to cause vomiting, whereas the Semont maneuver carried a relatively higher risk.

**Table 5:** League table for vomiting.

**Treatment**	**Epley**	**Semont**	**Brandt–Daroff**
Epley	NA	0.99 (0.52, 1.89)	0.94 (0.44, 1.93)
		⊕⊕⊕O Moderate	⊕⊕⊕O Moderate
Semont	1.02 (0.53, 1.93)	NA	0.96 (0.45, 1.97)
	⊕⊕⊕⊕O Moderate		⊕⊕⊕O Moderate
Brandt–Daroff	1.06 (0.52, 2.27)	1.04 (0.51, 2.25)	NA
	⊕⊕⊕O Moderate	⊕⊕⊕O Moderate	

Evidence quality: high (⊕⊕⊕⊕), moderate (⊕⊕⊕O), low (⊕⊕OO), very low (⊕OOO).

**Figure 9:**
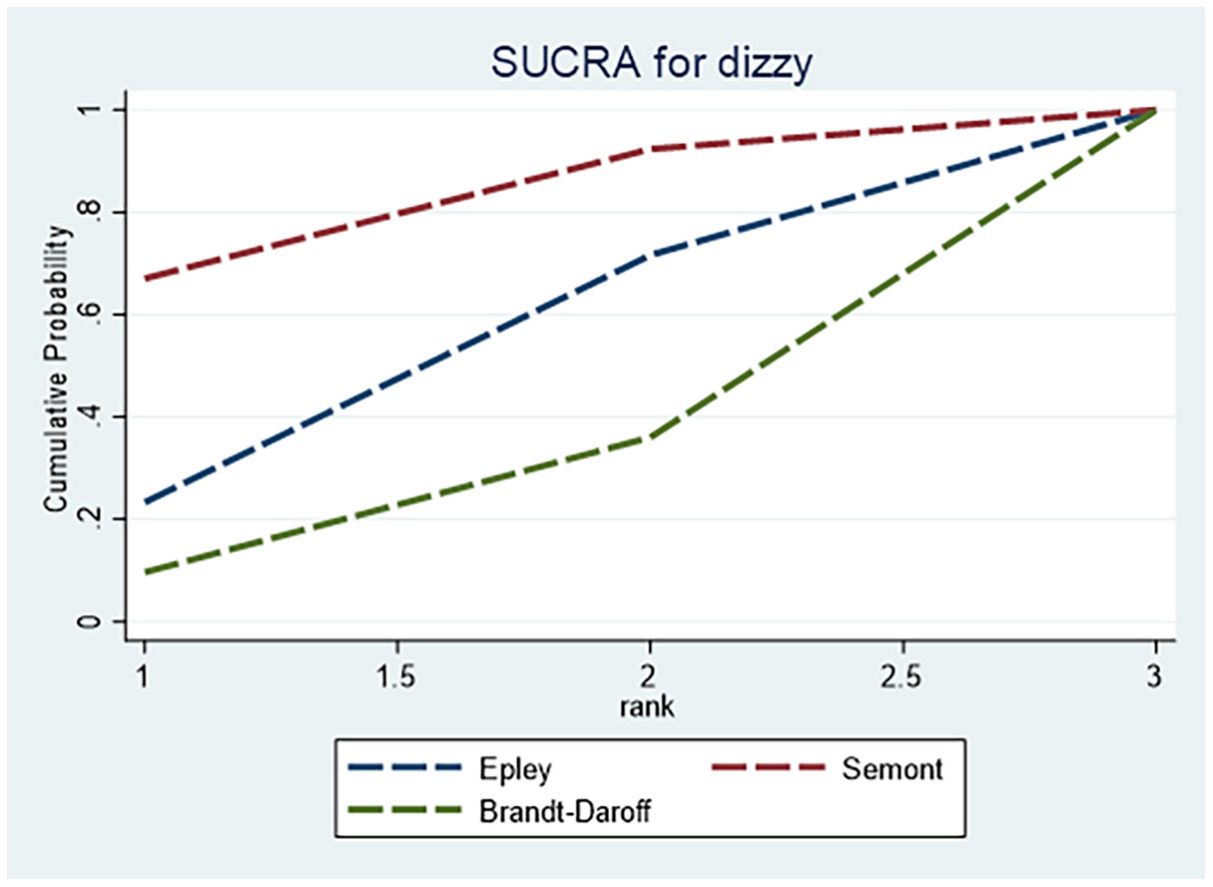
SUCRA ranking curve for dizziness.

For dizziness, Table 6 shows no statistically significant differences among the Epley, Semont and Brandt–Daroff maneuvers. Based on SUCRA values (Figure 9), the Epley maneuver showed the most favorable safety profile for dizziness, followed by the Semont and then the Brandt–Daroff maneuver.

**Table 6:** League table for dizziness.

**Treatment**	**Epley**	**Semont**	**Brandt–Daroff**
Epley	NA	0.91 (0.68, 1.22)	1.07 (0.81, 1.4)
		⊕⊕⊕O Moderate	⊕⊕⊕O Moderate
Semont	1.1 (0.82, 1.48)	NA	1.17 (0.88, 1.58)
	⊕⊕⊕O Moderate		⊕⊕⊕O Moderate
Brandt–Daroff	0.94 (0.71, 1.24)	0.86 (0.63, 1.14)	NA
	⊕⊕⊕O Moderate	⊕⊕⊕O Moderate	

Evidence quality: high (⊕⊕⊕⊕), moderate (⊕⊕⊕O), low (⊕⊕OO), very low (⊕OOO).

### 3.6 Heterogeneity and inconsistency assessment

Assessment of heterogeneity showed that *I*^2^ values for recurrence rate, cure rate, nausea, vomiting and dizziness were all below 50%, indicating low heterogeneity and stable findings. In contrast, the effectiveness outcome demonstrated high heterogeneity (*I*^2^ > 75%), indicating considerable variability across studies. Detailed results are presented in Supplementary Figures S1–6.

**Figure 10:**
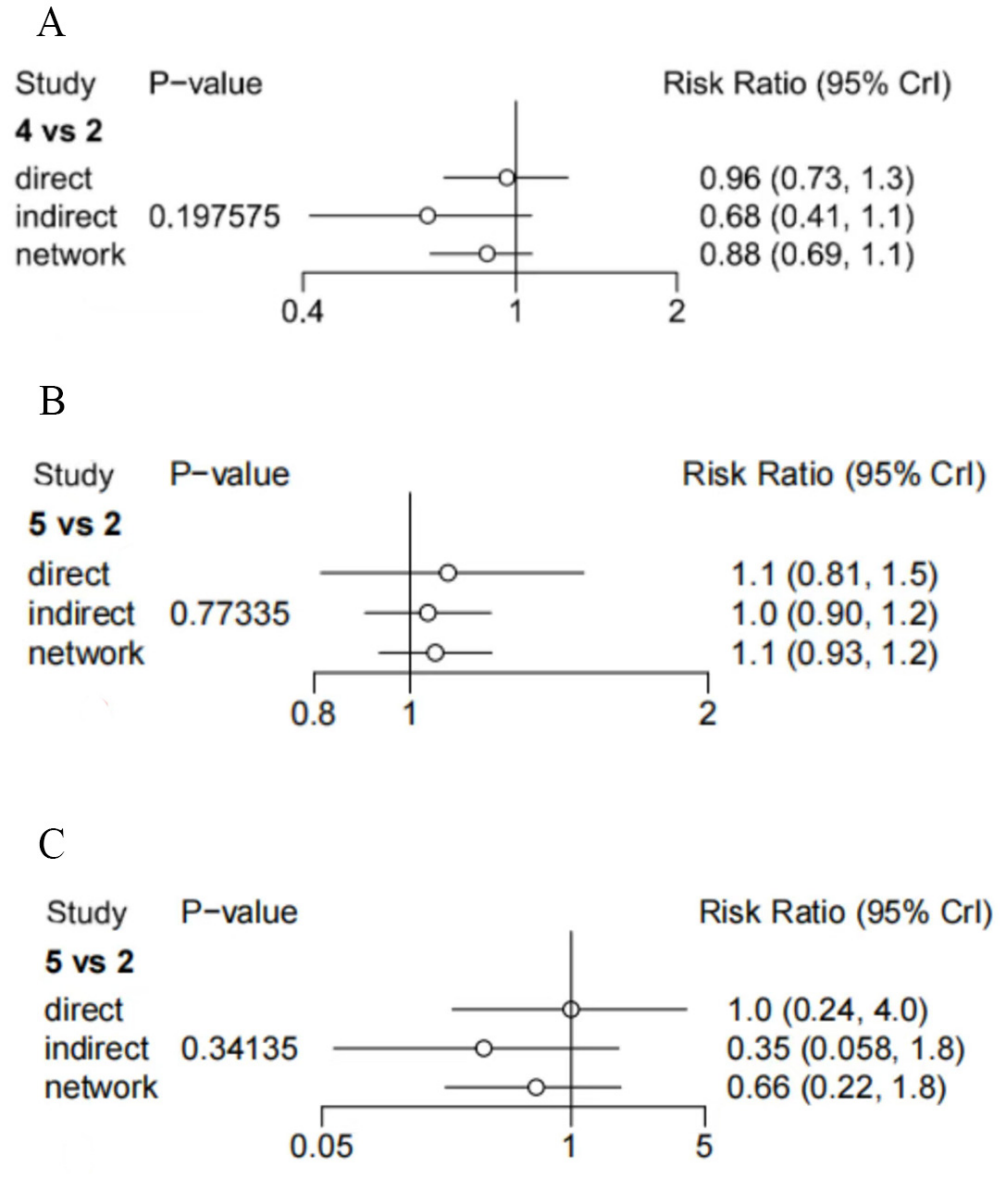
Results of inconsistency assessment (2: semont; 4 and 5: Gans), **(A)** efficiency; **(B)** cure rate; **(C)** recurrence rate.

Inconsistency was assessed using the node-splitting method for effectiveness, cure rate and recurrence rate separately. As shown in Figure 10, no statistically significant inconsistency between direct and indirect evidence was detected for any comparison, with all *P* values exceeding 0.05. These results indicate good statistical consistency within the network meta-analysis.

### 3.7 Meta-regression and publication bias

Meta-regression analyses were conducted to evaluate the influence of baseline characteristics, including publication year, sample size, sex ratio and mean patient age, on recurrence rate, effectiveness and cure rate. The 95% CIs of regression coefficients crossed zero for all covariates, indicating no statistically significant associations. Detailed data are presented in Supplementary Tables S3–5.

**Figure 11:**
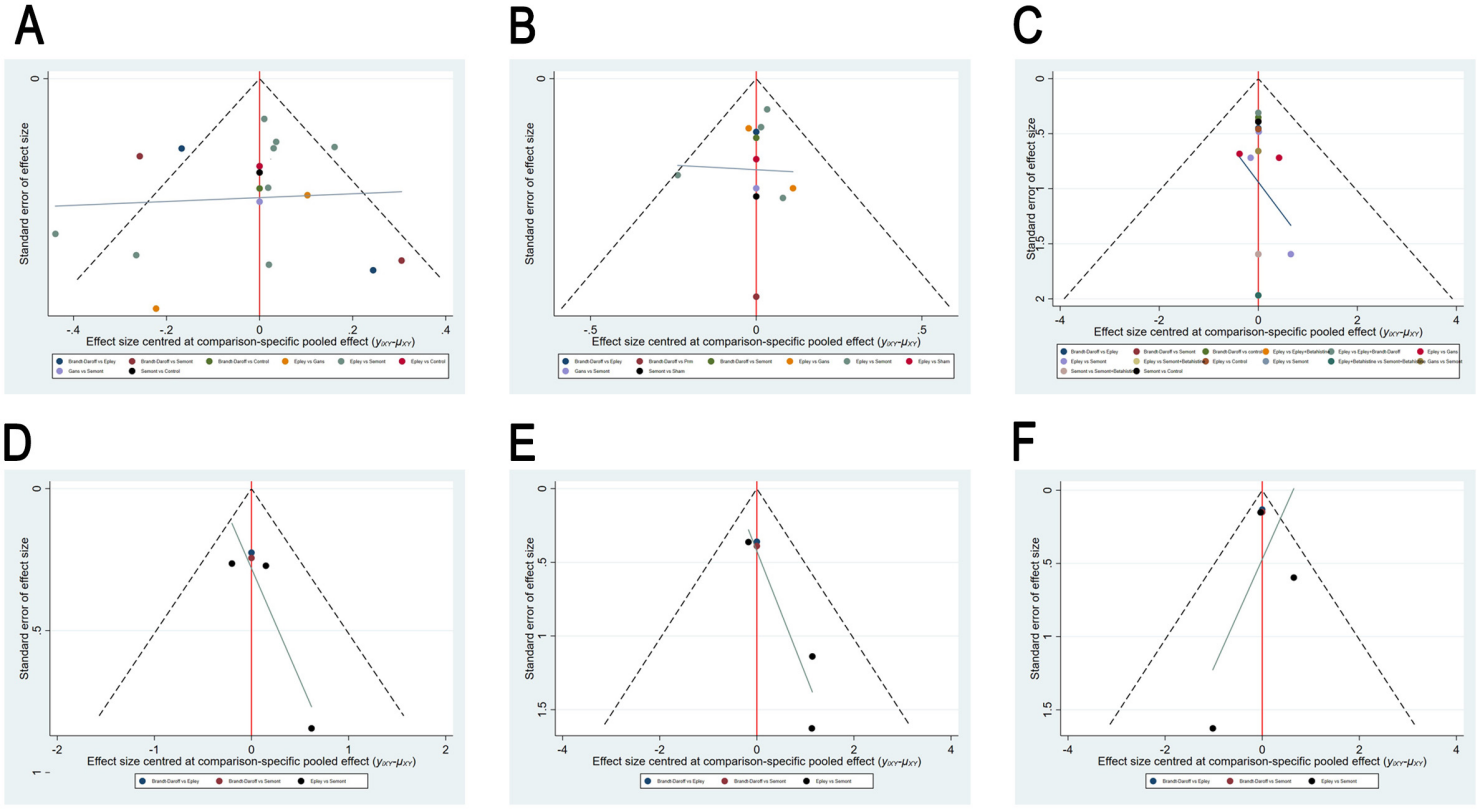
Funnel plots for publication bias assessment, **(A)** efficiency; **(B)** cure rate; **(C)** recurrence rate; **(D)** nausea; **(E)** vomiting; **(F)** dizziness.

Publication bias was assessed using funnel plots for effectiveness, cure rate, recurrence rate, nausea, vomiting and dizziness, as shown in Figure 11. The funnel plots showed symmetrical distribution of data points. Egger's test indicated a low likelihood of publication bias for effectiveness (*P* = 0.169), cure rate (*P* = 0.617) and recurrence rate (*P* = 0.775). For nausea, vomiting and dizziness, fewer than five studies were available (*n* < 5), precluding Egger's test.

### 3.8 Certainty of evidence

The GRADE assessment revealed considerable variation in evidence certainty across different outcomes, with most evidence rated as moderate or low quality, highlighting limitations of the current evidence base.

For the primary efficacy outcomes of cure rate and effectiveness, evidence certainty for key comparisons was relatively high. Comparisons between the Epley and Semont maneuvers, as well as their superiority over control or no-treatment conditions, were supported by moderate to high certainty evidence. These findings provide robust support for recommending these two maneuvers as first-line treatments for BPPV. Previous literature also supports the Epley and Semont maneuvers as first-line treatment options (4). However, for the Brandt–Daroff maneuver, the Gans maneuver and combined interventions, evidence certainty was predominantly low or very low, precluding reliable conclusions regarding their relative efficacy ranking or superiority over the established maneuvers. Prior research comparing the Semont-plus maneuver with the Epley maneuver did not include Brandt–Daroff or Gans maneuvers, indicating that comparative effectiveness among these techniques still requires high-quality evidence (4).

For safety outcomes including dizziness, vomiting and nausea, many studies did not systematically record adverse events or failed to distinguish pre-existing baseline symptoms (such as baseline dizziness or nausea) from treatment-induced adverse reactions (29). Evidence certainty for safety outcomes was generally lower. Although available data suggested no statistically significant differences among maneuvers for dizziness or vomiting, evidence quality ranged from moderate to low. For nausea specifically, all comparisons were supported only by low certainty evidence, indicating substantial uncertainty regarding potential differences among interventions. Conclusions regarding safety profiles of these maneuvers should therefore be interpreted with caution.

Furthermore, this study strictly adhered to pre-established inclusion and exclusion criteria throughout the literature screening process. Although several recently published RCTs have examined comparisons among different repositioning maneuvers, some were excluded for not reporting key outcome indicators or for duplicate publication of data. These decisions were made to ensure that the analysis focused specifically on direct comparisons of repositioning maneuvers, thereby minimizing the influence of confounding factors and enhancing internal validity. Future updates of this analysis may incorporate additional high-quality, rigorously designed RCTs to further expand the evidence network.

In summary, current evidence clearly supports the effectiveness of the Epley and Semont maneuvers as the cornerstone of BPPV treatment, although this evidence advantage is primarily based on a limited number of key comparisons. For other maneuvers, combined treatment regimens and safety outcomes, the existing evidence base remains weak. Future well-designed, large-sample RCTs are needed to strengthen evidence certainty and clarify the benefits and risks of these interventions (4).

## 4 Discussion

This network meta-analysis systematically evaluated the efficacy, safety and recurrence rates of multiple repositioning maneuvers for posterior canal BPPV. Twenty RCTs involving 2,089 patients were included, covering the Epley, Semont, Brandt–Daroff and Gans maneuvers. For effectiveness, the Epley maneuver performed best (SUCRA = 97.84%) and was significantly superior to the Semont maneuver, Brandt–Daroff exercises and control. For cure rate, both the Epley and Semont maneuvers ranked highest and demonstrated clear superiority over other interventions. For recurrence rate, evidence certainty was predominantly low or very low, precluding determination of which maneuver was most effective for reducing recurrence. Regarding safety, no statistically significant differences were observed among the Epley, Semont and Brandt–Daroff maneuvers for nausea, vomiting or dizziness, although SUCRA rankings suggested a trend toward better safety with the Epley maneuver.

This study included 20 RCTs conducted across multiple regions, including Germany, South Korea, India, North Korea, and the United States, encompassing populations from Asia, Europe, and North America, thereby conferring a degree of global representativeness to the findings. Although the geographical distribution of included studies showed some imbalance, with Asian studies predominating, the principal finding that the Epley maneuver demonstrated optimal effectiveness and cure rate, followed by the Semont maneuver, remained consistent across regional subgroups, with no significant geographical interaction effects detected. This consistency suggests that the advantages of the Epley and Semont maneuvers may be generalizable across diverse populations and clinical settings, strengthening the external validity and clinical applicability of these conclusions. Nevertheless, additional high-quality studies from underrepresented regions such as Africa and South America are warranted to further validate the global applicability of these repositioning maneuvers.

These findings are largely consistent with a recently published network meta-analysis (30), which similarly demonstrated that the Epley and Semont maneuvers ranked highest in both cure rate and effectiveness, with no significant difference between them. Both studies noted that the Brandt–Daroff maneuver was less effective in symptom relief and recurrence prevention, making it more suitable as an adjunctive or home-based training method. Notably, our findings also align with a long-term randomized controlled trial by Amor-Dorado et al. (20), which compared the modified Epley maneuver (particle repositioning maneuver, PRM) with Brandt–Daroff exercises for idiopathic posterior canal BPPV. That study found that PRM achieved significantly higher positional nystagmus resolution rates than Brandt–Daroff exercises in the short term (Day 7 and Month 1), whereas no difference in recurrence rates was observed over 48 months of follow-up; however, the interval to second recurrence was significantly prolonged in the PRM group. This real-world clinical evidence further supports the short-term efficacy advantage of the Epley maneuver and suggests a potential benefit in delaying recurrence, thereby strengthening the external validity and clinical relevance of the present network meta-analysis. We gratefully acknowledge all included randomized controlled trials for providing the essential direct and indirect comparative evidence underlying this study.

The Epley maneuver (modified canalith repositioning procedure) is currently the most widely used technique for posterior canal BPPV, and its effectiveness has been supported by multicenter studies (4, 7). Its mechanism is based on the anatomical characteristics of the posterior semicircular canal and the principles of otoconial dynamics. According to the theory of otoconial displacement, through a sequence of head positions (Dix–Hallpike position → head rotation toward the unaffected side → body rotation into the prone position → sitting up), the relative movement between the bony semicircular canal and endolymph, together with the inertial motion of solid otoconial particles within the endolymph, facilitates displacement of otoconia from the affected canal back into the utricle (4). Each position is maintained for 30–60 s to ensure adequate otoconial movement.

The Semont maneuver operates through a mechanism distinct from that of the Epley maneuver, primarily driven by inertial forces for otoconial clearance (12). The maneuver involves rapidly moving from a seated position to the affected side-lying position, followed by a swift transition to the opposite side, generating greater inertial forces through sudden acceleration (31). The key determinant of success is the speed of head and trunk movement. When the patient lies on the affected side, otoconia settle at the lowest point. During rapid movement to the opposite side, otoconia do not immediately fall to the ampullary end but instead exit through the upper opening at the apex due to inertia. If the movement is insufficiently rapid, otoconia fail to traverse the apex and return to the ampulla. This rapid movement may be particularly effective for adherent otoconia in cupulolithiasis.

The Brandt–Daroff exercises differ fundamentally in mechanism from the Epley and Semont maneuvers, operating through two principal components. The first involves dispersion and dissolution of otoconia: repeated positional changes such as rapid side-lying and sitting up generate mechanical forces that may dislodge otoconial fragments from the cupula and disperse them (1, 31), reducing vestibular stimulation and alleviating vertigo symptoms. The second component involves enhancement of central compensation: repetitive exposure to vertigo-provoking positional stimuli activates vestibular central compensation mechanisms (32), enabling gradual adaptation and reduced sensitivity to vertiginous stimuli, ultimately alleviating dizziness and balance impairment.

This network meta-analysis comprehensively compared the clinical efficacy and safety of multiple repositioning maneuvers for BPPV and provides clear support for the advantages of the Epley and Semont maneuvers in effectiveness and cure rate, offering robust evidence for their use as first-line treatments. These findings indicate that the Epley and Semont maneuvers should be prioritized in clinical practice, particularly for posterior canal BPPV, as they substantially improve treatment outcomes and may reduce recurrence risk. This analysis revealed relative limitations of the Brandt–Daroff method in both efficacy and supporting evidence. Although the Gans maneuver has been evaluated in relevant studies (33), the quantity and quality of available evidence remain insufficient to draw definitive conclusions. Oh et al. (31), suggesting that these maneuvers should be used with caution and that interventions supported by stronger evidence should be preferred.

## 5 Heterogeneity and limitations

A comprehensive heterogeneity assessment was performed for all included RCTs, encompassing primary efficacy outcomes (cure rate and effectiveness) and safety outcomes (recurrence rate, dizziness, vomiting and nausea). The results demonstrated marked variation in heterogeneity levels across different outcomes. For most direct comparisons and network estimates, recurrence rate, cure rate, dizziness, vomiting and nausea exhibited very low heterogeneity, indicating that effect sizes from different studies were highly consistent when evaluating the same intervention. The observed differences were likely attributable to random variation rather than true inter-study differences.

Pronounced heterogeneity was observed for effectiveness, a key efficacy outcome. This heterogeneity may be attributed to two major sources. The first relates to inconsistent baseline population characteristics across studies. Age distribution varied markedly, with mean ages ranging from 26.5 ± 14.8 years to 65.8 ± 8.9 years; differences in age may influence tolerance to repositioning maneuvers and the capacity for central compensation. The proportion of male participants also differed considerably (approximately 25% to nearly 50%), and sex may influence vestibular compensation rates. Furthermore, disease duration and BPPV subtype were not uniformly defined; although most studies focused on posterior canal BPPV, several did not specify whether cases were acute or chronic or whether multicanal involvement was excluded, affecting comparability across trials. The second source of heterogeneity relates to variation in intervention protocols. Some studies employed combined therapy with adjunctive medication such as betahistine, while others used single maneuvers alone; pharmacological adjuncts may alter treatment effectiveness. Control conditions were also inconsistent, including no treatment, sham procedures or alternative active maneuvers, which affected the baseline for relative effectiveness assessment.

Regarding methodological limitations, differences in the operational principles and execution of repositioning maneuvers may contribute to inconsistent treatment effects. The Epley maneuver uses sequential head position changes to guide otoconia out of the posterior semicircular canal, whereas the Semont maneuver relies on rapid lateral movements. Technical deviations such as variation in head–neck angles or movement speed may influence repositioning success. The Brandt–Daroff habituation exercises operate through repeated induction of vertigo to enhance central compensation (1), a mechanism differing from the preceding maneuvers; however, patient self-administration introduces adherence challenges. The Gans maneuver incorporates dynamic assessment and individualized repositioning steps, further increasing procedural complexity. Overall, treatment effectiveness is highly dependent on operator proficiency and experience (31).

Regarding study populations, although this study incorporated data from multiple countries, studies from Asian regions constituted a disproportionately large share, with Europe, North America, and other regions underrepresented, potentially limiting the global generalizability of the conclusions. Future geographically balanced, rigorously designed multicenter RCTs are needed to further validate the consistency of repositioning maneuver efficacy across diverse populations.

Technological innovations including fully automated mechanical repositioning devices have expanded clinical possibilities (34). Such systems offer precise control of head rotation, posture transitions and angular adjustments across multiple axes, enabling individualized modifications of repositioning protocols for clinicians, enhancing clinical quality control, reducing dependency on operator skill levels and improving feasibility for implementation in primary healthcare settings.

Standardization of maneuver performance and consistency evaluation is therefore essential. Standardized video instructional materials should be developed, and multicenter operator training with competency assessment should be organized to ensure consistent execution of key procedural steps. For habituation exercises such as the Brandt–Daroff method requiring patient self-administration, supportive tools or digital guidance programs should be developed to improve adherence. Future studies should systematically document and report operator experience, number of repositioning attempts and use of auxiliary equipment to enhance reproducibility and transparency.

## 5 Conclusion

This network meta-analysis systematically evaluated the efficacy and safety of multiple repositioning maneuvers, including the Epley, Semont, Brandt–Daroff and Gans maneuvers, for posterior canal BPPV (pc-BPPV). For short-term efficacy, the Epley maneuver achieved the highest effectiveness and cure rates, followed by the Semont maneuver; both were significantly superior to the Brandt–Daroff maneuver and control, and should be recommended as first-line repositioning strategies for pc-BPPV. It should be emphasized that SUCRA rankings reflect only the relative standing of interventions within the available evidence and do not indicate effect size magnitude or evidence quality; clinical decision-making should therefore integrate specific effect estimates with individual patient circumstances. Regarding safety, no statistically significant differences were observed among the major maneuvers for nausea, vomiting or dizziness, although SUCRA rankings indicated a more favorable safety trend for the Epley maneuver. For recurrence rate, the overall certainty of evidence was low, and no optimal intervention could be clearly identified. Evidence supporting the Brandt–Daroff and Gans maneuvers remained limited. For the Gans maneuver specifically, although relevant studies exist, the paucity of high-quality RCTs enabling direct comparisons with other maneuvers has resulted in predominantly low or very low certainty of evidence according to GRADE assessment; its precise efficacy ranking relative to the Epley or Semont maneuvers therefore awaits confirmation through additional rigorously designed studies. The substantial heterogeneity observed in effectiveness may be related to differences in population characteristics, intervention details and inconsistent outcome assessment standards across studies. In summary, this study provides evidence-based support for the advantages of the Epley and Semont maneuvers in the treatment of pc-BPPV, while also highlighting limitations in current evidence regarding recurrence outcomes and secondary maneuvers. Future research should prioritize high-quality, standardized RCTs, with particular attention to long-term recurrence outcomes, procedural standardization and efficacy differences across diverse populations, to further enhance the precision and generalizability of repositioning treatments for pc-BPPV.

## Data Availability

The original contributions presented in the study are included in the article/Supplementary material, further inquiries can be directed to the corresponding author.

## Supplementary material

Supplementary Table S1 Search strategy.

Supplementary Table S2 Basic characteristics of included studies.

Supplementary Table S3 Regression analysis of efficacy rate.

Supplementary Table S4 Regression analysis of the cure rate.

Supplementary Table S5 Regression analysis of recurrence rate.

Supplementary Figure S1 Heterogeneity test for efficacy.

Supplementary Figure S2 Heterogeneity test for cure rate.

Supplementary Figure S3 Heterogeneity test for recurrence rate.

Supplementary Figure S4 Heterogeneity test for nausea.

Supplementary Figure S5 Heterogeneity test for vomiting.

Supplementary Figure S6 Heterogeneity test for dizziness.
