# Supplementary material for: Comparative efficacy and safety of repositioning maneuvers for posterior canal benign paroxysmal positional vertigo: a network meta-analysis
Source: Front Neurol. 2026 Feb 2;17:1762375. doi: 10.3389/fneur.2026.1762375 (PMC12907698; doi:10.3389/fneur.2026.1762375)
Supplement: Supplementary Table S1 — Search strategy. [file Table_1.docx]

**Supplementary Materials**

**Supplementary Table S1** **Search Strategy**

| Pubmed | | |
| --- | --- | --- |
| # | Query | Results |
| 1 | Benign Paroxysmal Positional Vertigo[MeSH Terms] | 1,395 |
| 2 | "benign paroxysmal postural vertigo"[Title/Abstract] OR "benign postural paroxysmal vertigo"[Title/Abstract] OR "Benign Recurrent Vertigo*"[Title/Abstract] OR "BPPV"[Title/Abstract] OR "familial vestibulopath*"[Title/Abstract] OR "paroxysmal positional vertigo"[Title/Abstract] OR "positional paroxysmal vertigo"[Title/Abstract] | 3,124 |
| 3 | #1 OR #2 | 3,225 |
| 4 | "epley"[Title/Abstract] OR "Semont"[Title/Abstract] OR "Brandt Daroff"[Title/Abstract] OR "Gufoni"[Title/Abstract] OR "Gans"[Title/Abstract] | 1,850 |
| 5 | #3 AND #4 | 526 |

| Embase | | |
| --- | --- | --- |
| # | Query | Results |
| 1 | 'benign paroxysmal positional vertigo'/exp | 684 |
| 2 | 'benign paroxysmal postural vertigo':ab,ti,kw OR 'benign postural paroxysmal vertigo':ab,ti,kw OR 'benign recurrent vertigo*':ab,ti,kw OR 'bppv':ab,ti,kw OR 'familial vestibulopath*':ab,ti,kw OR 'paroxysmal positional vertigo':ab,ti,kw OR 'positional paroxysmal vertigo':ab,ti,kw | 2182 |
| 3 | #1 OR #2 | 4624 |
| 4 | 'epley':ab,ti,kw OR 'semont':ab,ti,kw OR 'brandt daroff':ab,ti,kw OR 'gufoni':ab,ti,kw OR 'gans':ab,ti,kw | 3842 |
| 5 | #3 AND #4 | 3601 |

| Cochrane Library | | |
| --- | --- | --- |
| # |  |  |
| 1 | MeSH descriptor: [Benign Paroxysmal Positional Vertigo] explode all trees | 168 |
| 2 | (‘benign paroxysmal postural vertigo’ OR ‘benign postural paroxysmal vertigo’ OR ‘Benign Recurrent Vertigo*’ OR ‘BPPV’ OR 'familial vestibulopath*’ OR ‘paroxysmal positional vertigo’ OR ‘positional paroxysmal vertigo’):ab,ti,kw | 543 |
| 3 | #1OR#2 | 543 |
| 4 | (‘epley’ OR ‘Semont’ OR ‘Brandt Daroff’ OR ‘Gufoni’ OR ‘Gans’):ab,ti,kw | 277 |
| 5 | #3AND#4 | 242 |

| Web of Science | | |
| --- | --- | --- |
| # | Query | Results |
| 1 | TS=((benign paroxysmal postural vertigo) OR (benign postural paroxysmal vertigo) OR (Benign Recurrent Vertigo*) OR (BPPV) OR (familial vestibulopath*) OR (paroxysmal positional vertigo) OR (positional paroxysmal vertigo)) | 140 |
| 2 | TS=((epley) OR (Semont) OR (Brandt Daroff) OR (Gufoni) OR (Gans)) | 337 |
| 3 | #2 AND #1 | 9 |

**Supplementary Table S2 Basic characteristics of included studies**

| ID | Author | Year | Region | Sample size | Interventions | Number of people | Gender (Male/Female) | Age |
| --- | --- | --- | --- | --- | --- | --- | --- | --- |
| 1 | Yan-Xing Zhang | 2016 | Shaoxing city, China | 168 | Epley technique | 45 | 15/30 | 51.38±10.66 |
|  |  |  |  |  | Semont technique | 43 | 14/29 | 49.79±13.30 |
|  |  |  |  |  | Brandt-Daroff technique | 40 | 15/25 | 52.60±13.94 |
|  |  |  |  |  | Blank control | 40 | 17/23 |  |
| 2 | Mansi A R Venkatramanan | 2023 | India | 28 | Epley technique | 14 | Total 15/13 | Total 52.5 |
|  |  |  |  |  | Semont technique | 14 |  |  |
| 3 | Lakshmi Unnikrishnan | 2023 | India | 100 | Epley technique | 50 | 19/31 | 59±9.16 |
|  |  |  |  |  | Semont technique | 50 | 18/32 |  |
| 4 | Mahesh G. Santhraya | 2021 | India | 30 | Semont technique | 15 | 5/10 | 48.1 |
|  |  |  |  |  | Epley technique | 15 | 7/8 |  |
| 5 | Alia Saberi | 2017 | Germany | 60 | Epley technique | 30 | 7/23 | 46.9±13.4 |
|  |  |  |  |  | Gans technique(G) | 30 | 8/22 | 46.7±7.5岁 |
| 6 | A. Radtke, MD | 2004 | Germany | 70 | Epley technique | 37 |  |  |
|  |  |  |  |  | Semont technique | 33 |  |  |
| 7 | Shruti V. | 2024 | India | 90 | Epley technique | 30 | Total 41/49 | 45.35±10.96 |
|  |  |  |  |  | Semont technique | 30 |  |  |
|  |  |  |  |  | Gans technique | 30 |  |  |
| 8 | Prasun Mishra1 | 2023 | India | 200 | Epley technique | 100 | Total 71/129 | 45.45 |
|  |  |  |  |  | Semont technique | 100 |  | 45.83 |
| 9 | Jong Dae Lee a | 2014 | North Korea | 99 | Epley technique | 36 | 45956 | 57.3±14.1 |
|  |  |  |  |  | Semont technique | 32 | 45897 | 56.9±14.7 |
|  |  |  |  |  | sham technique | 31 | 45774 | 56.8±12.5 |
| 10 | Ramesh Guna keerthana | 2021 | India | 108 | Epley technique | 54 | 25/29 | 26.5±14.8 |
|  |  |  |  |  | Semont technique | 54 | 26/28 |  |
| 11 | Ashok Kumar Gupta1 | 2019 | India | 90 | Epley technique | 30 | Total 31/59 | 49.96±13.96 |
|  |  |  |  |  | Semont technique | 30 |  |  |
|  |  |  |  |  | Brandt-Daroff technique | 30 |  |  |
| 12 | Bandana Thakur | 2024 | Germany | 170 | Epley technique | 85 | Total 82/88 | 47.14±14.71 |
|  |  |  |  |  | Semont technique | 85 |  |  |
| 13 | Michael Strupp | 2023 | America | 195 | Epley technique | 97 | 30/67 | 60.9±13.8 |
|  |  |  |  |  | Semont technique | 98 | 40/58 | 64.4±13.9 |
| 14 | Chayada Sinsamutpadung | 2021 | Thailand | 80 | Epley technique | 40 | 11/29 | 61.73±11.28 |
|  |  |  |  |  | Semont technique | 40 | 12/28 | 61.73±13.93 |
| 15 | Sheetal | 2023 | Thailand | 30 | Semont technique+ Brandt-Daroff | 16 |  | 45.19±12.89 |
|  |  |  |  |  | Brandt-Daroff | 14 |  | 42.79±13.66 |
| 16 | K. Ravi | 2023 | India | 94 | Epley technique | 47 | 21/26 |  |
|  |  |  |  |  | Epley technique+Brandt–Daroff | 47 | 22/25 |  |
| 17 | Neetu R. Dhiman | 2023 | India | 234 | Epley technique | 118 | 44/74 | 43.9±12.6 |
|  |  |  |  |  | Gans technique | 116 | 48/68 | 48.4±14 |
| 18 | Faizah Ashfah Latief Deva | 2024 | India | 100 | Epley technique | 50 | Total 41/59 | 46.3±18.5 |
|  |  |  |  |  | Semont technique | 50 |  | 46.1±17.8 |
| 19 | Seo-Young Choi | 2020 | Republic of Korea | 62 | Epley technique | 29 | 8/21 | 65.8 ± 8.9 |
|  |  |  |  |  | Brandt-Daroff | 33 | 8/25 | 64.2 ± 12.0 |
| 20 | Juan Carlos Amor-Dorado | 2012 | Spain | 81 | PRM | 41 | 16/25 | 58.8±11.7 |
|  |  |  |  |  | Brandt-Daroff | 40 | 25/15 | 59.4±14.2 |

**Supplementary Table S3** Regression analysis of efficacy rate

| Year | Coefficient | 95%CI |
| --- | --- | --- |
| Epley | -0.1698 | -2.22329 to 1.6534 |
| Semont | -0.2364 | -2.33930 to 1.5566 |
| Brandt-Daroff | -0.4555 | -10.59656 to 5.3980 |
| Gans | 0.4305 | -1.52393 to 2.8129 |
| Epley+Brandt–Daroff | -2.8738 | -45.33543 to 6.3873 |
|  |  |  |
| Sample size | Coefficient | 95%CI |
| Epley | -0.3028 | -2.83177 to 1.6532 |
| Semont | -0.1591 | -2.74208 to 1.8883 |
| Brandt-Daroff | -0.1051 | -10.49910 to 7.0128 |
| Gans | 0.6083 | -2.49838 to 5.5858 |
| Epley+Brandt–Daroff | 1.1788 | -12.84622 to 14.3064 |
|  |  |  |
| Gender ratio | Coefficient | 95%CI |
| Epley | -0.34463 | -2.65202 to 1.6288 |
| Semont | -0.07192 | -2.61372 to 2.4449 |
| Brandt-Daroff | 0.12654 | -9.16908 to 12.7480 |
| Gans | 0.42617 | -1.67274 to 3.0437 |
| Epley+Brandt–Daroff | 0.32419 | -6.54417 to 10.5784 |
|  |  |  |
| Age | Coefficient | 95%CI |
| Epley | 0.02662 | -2.0880 to 2.2941 |
| Semont | 0.24941 | -1.6768 to 2.5007 |
| Brandt-Daroff | -0.26971 | -11.3807 to 6.5761 |
| Gans | -0.62136 | -6.0309 to 2.7321 |
| Epley+Brandt–Daroff | 1.13307 | -5.3563 to 18.7755 |

**Supplementary Table S4 Regression analysis of the cure rate**

| Year | Coefficient | 95%CI |
| --- | --- | --- |
| Epley | -1.68224 | -8.30660 to 3.4846 |
| Semont | 0.40553 | -5.67974 to 5.8249 |
| Brandt-Daroff | 1.47768 | -12.65738 to 24.2951 |
| Gans | -0.20097 | -12.16697 to 9.2293 |
| sham | 1.38527 | -7.81857 to 17.9577 |
| PRM | -0.04869 | -17.94313 to 22.7869 |
|  |  |  |
| Sample size | Coefficient | 95%CI |
| Epley | 2.52443 | -3.19986 to 15.9739 |
| Semont | -3.40516 | -17.55937 to 4.8912 |
| Brandt-Daroff | -3.90683 | -51.96747 to 19.2466 |
| Gans | 2.32408 | -3.61461 to 18.2793 |
| sham | -1.14478 | -24.80918 to 17.0272 |
| PRM | 3.40634 | -14.65302 to 38.9240 |
|  |  |  |
| Gender ratio | Coefficient | 95%CI |
| Epley | -0.2169 | -5.0461 to 5.50208 |
| Semont | 0.809 | -4.0340 to 7.03830 |
| Brandt-Daroff | -0.2515 | -33.2019 to 19.98087 |
| Gans | -0.0499 | -13.4589 to 12.49698 |
| sham | 3.0258 | -9.2380 to 22.44294 |
| PRM | 6.2694 | -24.5930 to 86.42904 |
|  |  |  |
| Age | Coefficient | 95%CI |
| Epley | 0.16804 | -5.11876 to 5.766786 |
| Semont | -0.05672 | -5.34126 to 5.808271 |
| Brandt-Daroff | 3.11498 | -11.11578 to 34.269579 |
| Gans | 0.47486 | -17.08528 to 24.174216 |
| sham | 8.1011 | -13.46835 to 87.960281 |
| PRM | -5.0405 | -45.52792 to 10.347274 |

**Supplementary Table S5 Regression analysis of recurrence rate**

| Year | Coefficient | 95%CI |
| --- | --- | --- |
| Epley | -0.1698 | -2.22329 to 1.6534 |
| Semont | -0.2364 | -2.33930 to 1.5566 |
| Brandt-Daroff | -0.4555 | -10.59656 to 5.3980 |
| Gans | 0.4305 | -1.52393 to 2.8129 |
| Epley+Brandt–Daroff | -2.8738 | -45.33543 to 6.3873 |
|  |  |  |
| Sample size | Coefficient | 95%CI |
| Epley | -0.3028 | -2.83177 to 1.6532 |
| Semont | -0.1591 | -2.74208 to 1.8883 |
| Brandt-Daroff | -0.1051 | -10.49910 to 7.0128 |
| Gans | 0.6083 | -2.49838 to 5.5858 |
| Epley+Brandt–Daroff | 1.1788 | -12.84622 to 14.3064 |
|  |  |  |
| Gender ratio | Coefficient | 95%CI |
| Epley | -0.34463 | -2.65202 to 1.6288 |
| Semont | -0.07192 | -2.61372 to 2.4449 |
| Brandt-Daroff | 0.12654 | -9.16908 to 12.7480 |
| Gans | 0.42617 | -1.67274 to 3.0437 |
| Epley+Brandt–Daroff | 0.32419 | -6.54417 to 10.5784 |
|  |  |  |
| Age | Coefficient | 95%CI |
| Epley | 0.02662 | -2.0880 to 2.2941 |
| Semont | 0.24941 | -1.6768 to 2.5007 |
| Brandt-Daroff | -0.26971 | -11.3807 to 6.5761 |
| Gans | -0.62136 | -6.0309 to 2.7321 |
| Epley+Brandt–Daroff | 1.13307 | -5.3563 to 18.7755 |


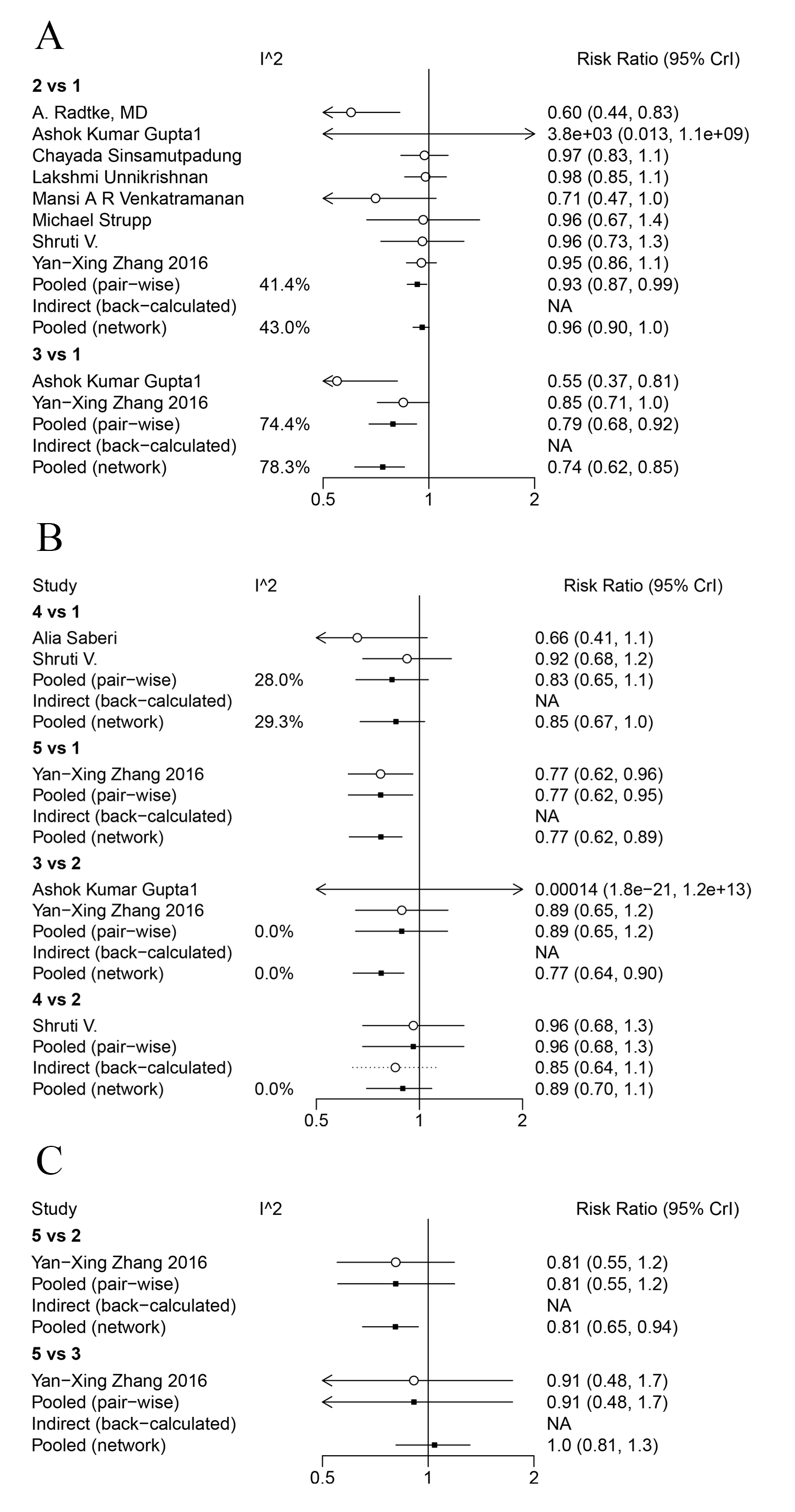


**Supplementary Figure S1** Heterogeneity Test for Efficacy

**
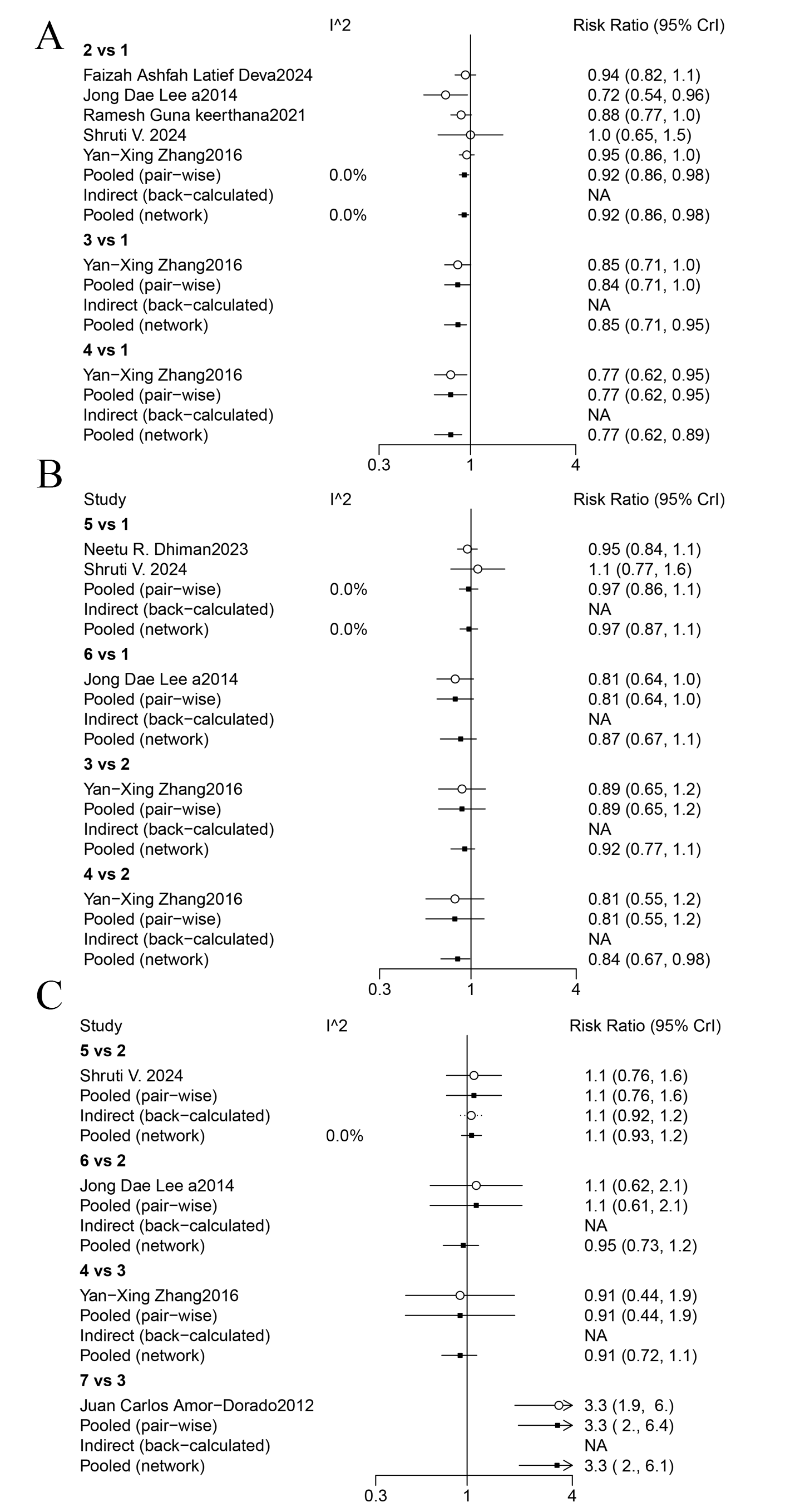
**

**Supplementary Figure S2** Heterogeneity Test for Cure Rate


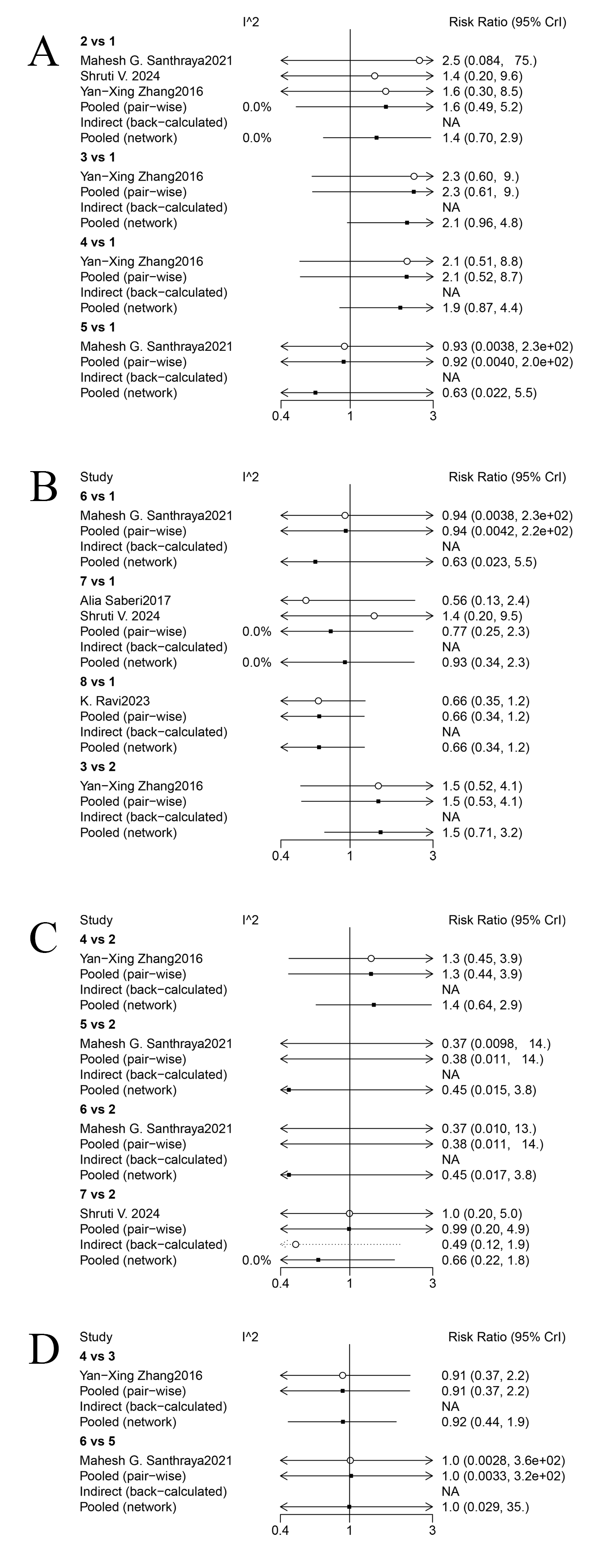


**Supplementary Figure S3 Heterogeneity Test for recurrence Rate**

**
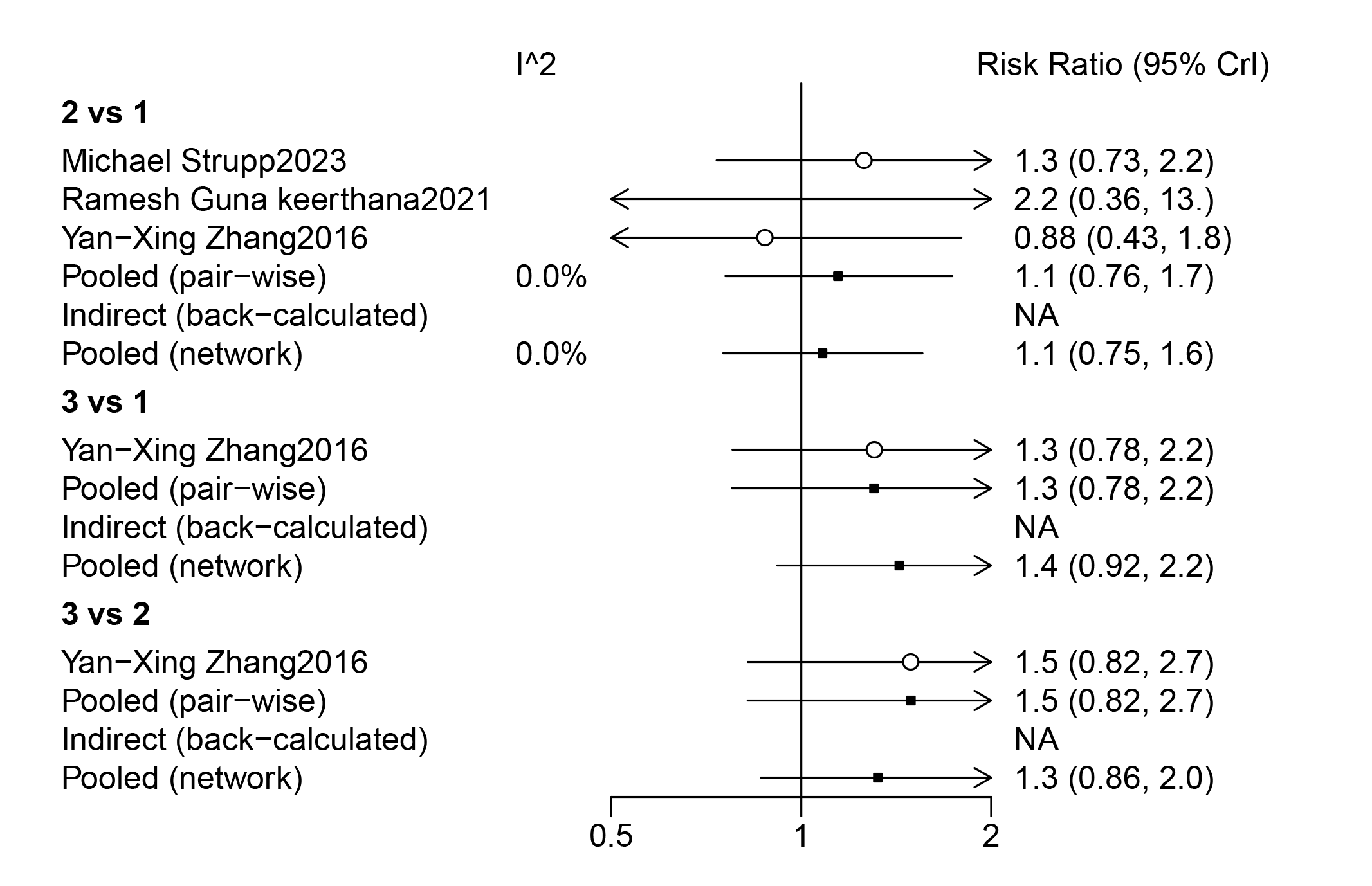
**

**Supplementary Figure S4 Heterogeneity Test for Nausea**

**
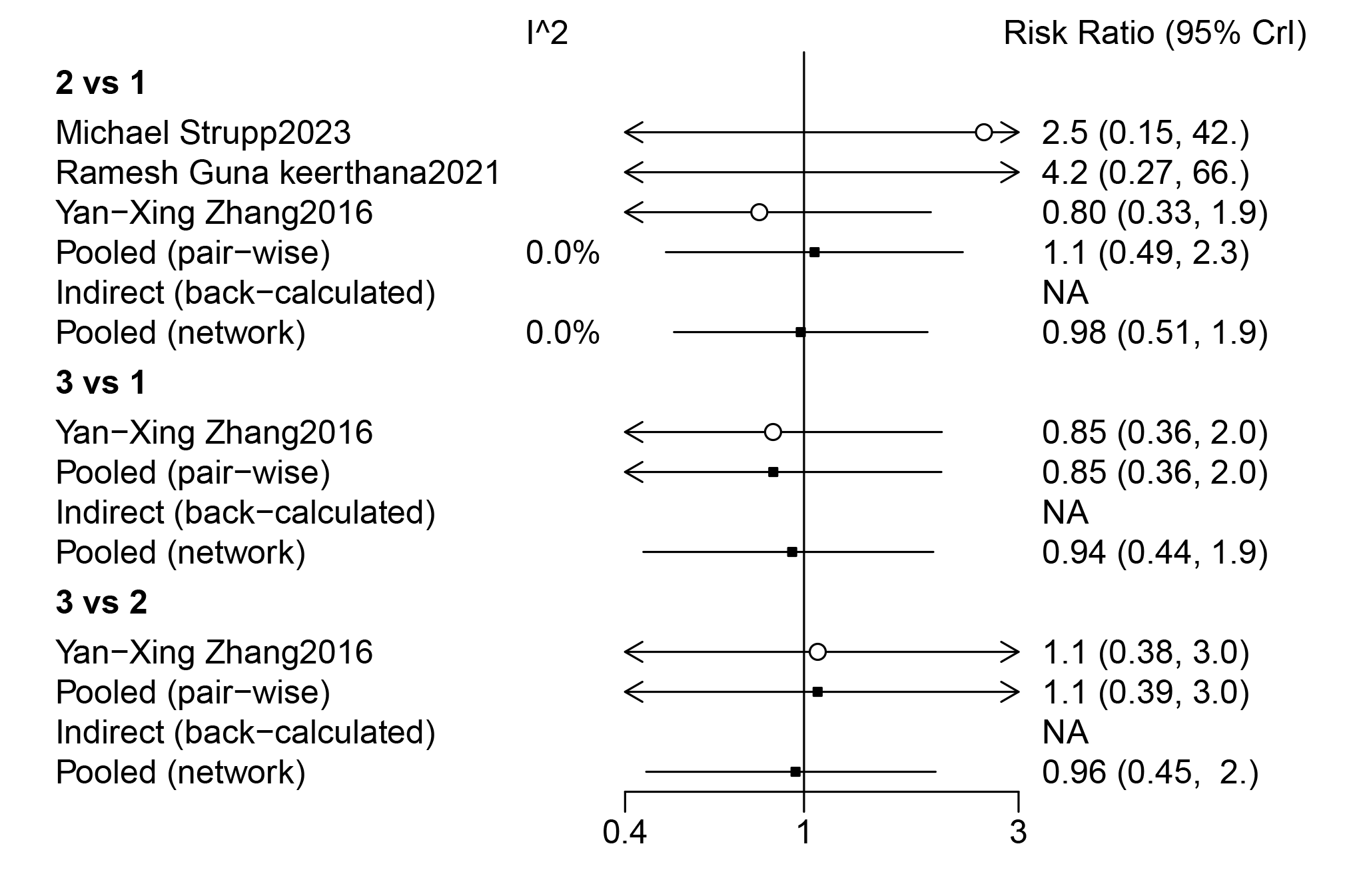
**

**Supplementary Figure S5 Heterogeneity Test for Vomiting**


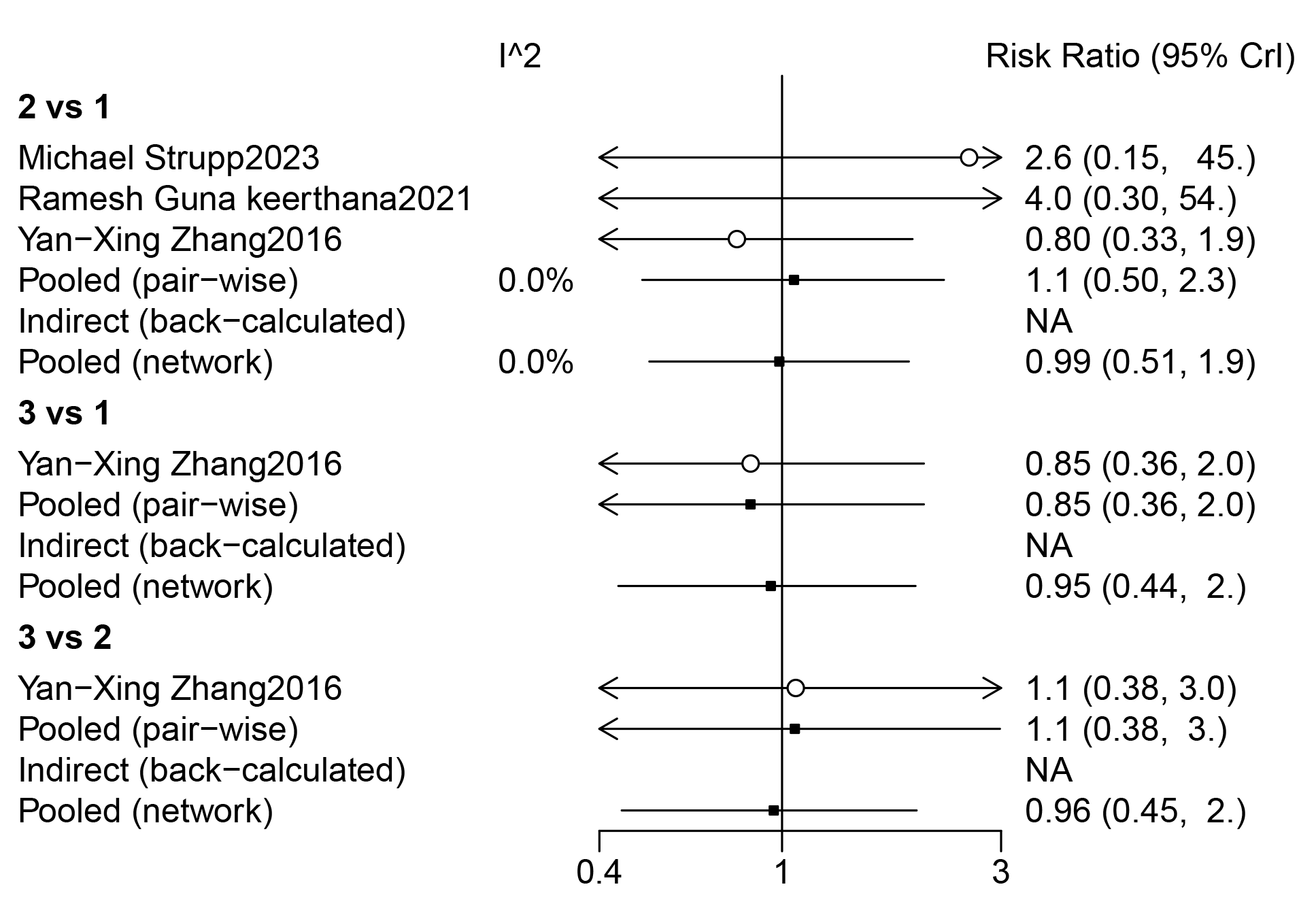


**Supplementary Figure S6 Heterogeneity Test for Dizziness**
